# Validation of Filament Materials for Injection Moulding 3D-Printed Inserts Using Temperature and Cavity Pressure Simulations

**DOI:** 10.3390/ma19020369

**Published:** 2026-01-16

**Authors:** Daniele Battegazzore, Alex Anghilieri, Giorgio Nava, Alberto Frache

**Affiliations:** 1 Dipartimento di Scienza Applicata e Tecnologia, Politecnico di Torino, Alessandria Site, V.le Teresa Michel 5, 15121 Alessandria, Italy; daniele.battegazzore@polito.it; 2 Moldex3D Italia, Larco Caleotto 30, 23900 Lecco, Italy; alexanghilieri@moldex3d.it (A.A.); giorgionava@moldex3d.it (G.N.)

**Keywords:** material extrusion 3D printing, mold inserts, simulation, injection molding, thermo-mechanical properties, thermal conductivity

## Abstract

**Highlights:**

**What are the main findings?**

- Simulation and rapid tests effectively screen MEX filaments for IM inserts.
- Nine filaments were suitable for LDPE moulding and five for PP conditions.
- Simulated temperatures closely matched thermal camera measurements.

**What are the implications of the main findings?**

- MEX inserts are viable for low-volume injection moulding applications.
- Combining testing and simulation reduces cost and development time.
- Design compensation can address dimensional limits of some filaments.

**Abstract:**

Using additive manufacturing for the design of inserts in injection moulding (IM) offers advantages in product development and customization. However, challenges related to operating temperature and mechanical resistance remain. This article presents a systematic screening methodology to evaluate the suitability of materials for specific applications. Ten commercial Material Extrusion (MEX) filaments were selected to produce test samples. Moldex3D simulation software was employed to model the IM process using two thermoplastics and to determine the temperature and pressure conditions that the printed inserts must withstand. Simulation results were critically interpreted and cross-referenced with the experimental material characterisations to evaluate material suitability. Nine of the ten MEX materials were suitable for IM with LDPE, and five with PP. Dimensional assessments revealed that six insert solutions required further post-processing for assembly, while three did not. All of the selected materials successfully survived 10 injection cycles without encountering any significant issues. The simulation results were validated by comparing temperature data from a thermal imaging camera during IM, revealing only minor deviations. The study concludes that combining targeted material characterization with CAE simulation provides an effective and low-cost strategy for selecting MEX filaments for injection moulding inserts, supporting rapid tooling applications in niche production.

## 1. Introduction

The mass production of plastic objects has prompted technologists to design and manufacture moulds capable of producing millions, or even billions, of identical parts through injection moulding. However, the rapid evolution of products and the growing demand for unique or customised items has drawn attention to alternative manufacturing technologies, such as additive manufacturing (AM)—commonly known as 3D printing [1,2]. In this way, the respective advantages and disadvantages of the two opposing technologies of subtractive and additive manufacturing are revealed [3,4]. In general, an additive solution can be used for rapid prototyping or to produce a small number of complicated objects quickly and economically, whereas a subtractive solution requires a large investment of time and money in the production of tools and moulds that can be used almost indefinitely [5]. Additive manufacturing (AM) parts can be produced to confirm the fit, form and function before the creation of an expensive metal mould. They can also be used as finished pieces when creating a mould would be too costly. However, not all materials can be used in AM processes or are available for them [6].

In this scenario, a middle-ground solution is to create moulds or tools using AM for use in the IM process, combining the advantages of both production technologies while limiting their disadvantages [7]. This allows inserts with specific geometries and/or functions to be built inside conventional metal moulds for niche production. Furthermore, thanks to the versatility of AM, these inserts could contain sophisticated cooling channels (conformal cooling), which are not feasible with a subtractive manufacturing process [4,8,9,10,11,12].

This approach has been explored in several industrial contexts where rapid turnarounds and highly customised or short production runs (e.g., pre-series parts, functionality trials, or specialised components for automotive, medical or consumer electronics markets) are important before investing in expensive hardened tool steel moulds. For example, using AM tools can reduce prototyping lead times from weeks/months to days, supporting quick design iteration cycles and enabling early detection of manufacturing issues, which is particularly valuable in competitive product development environments [13].

The production of such ‘hybrid’ moulds using AM offers several well-known benefits, particularly when thermoplastic materials are used [14]. On the other hand, heat dissipation, compression strength at different temperatures, and the coefficient of thermal expansion are critical properties that generally have an adverse effect on such hybrid AM moulds [1,2,3,9,15,16,17,18].

Conventional moulds made from hardened steel can generally dissipate thermal energy from polymer melts quickly (e.g., 16–20 W/m K for X40Cr14), whereas polymer-based AM inserts are usually thermal insulators (0.15–0.35 W/m K) [19]. Furthermore, the different coefficients of linear thermal expansion (CLTE) of coupled metal and polymeric inserts could cause problems with interference or fatigue failure, as well as dimensional deviation of the moulded parts. Finally, significant pressures are applied to the material in the molten state during the injection and packing phases of the injection moulding process. When the mould cavity is filled, melt pressure generates compressive stress on the various mould elements, including the inserts [20]. For this reason, the insert materials must be able to withstand such loads, which will occur at higher temperatures rather than room temperature, since the heating and cooling IM cycle generates temperature transients. Selecting suitable polymer-based MEX materials for these conditions therefore remains a significant research gap in the field [1,3,4,18,21].

Stereolithography (SLA), which belongs to the Vat Polymerisation category, has been extensively investigated as a technology for producing hybrid moulds, but it is still limited by the cost of the resin material and equipment, and it is made of thermoset plastics [4,7,17,22,23]. Krizsma et al. investigated a mould insert made from polyamide powder using Powder Bed Fusion (PBF) technology, which belongs to the Selective Laser Sintering (SLS) category. However, they did not have the opportunity to use materials containing fillers, which could provide significant advantages in terms of stiffness and thermal conductivity for the inserts [18].

On the other hand, Material Extrusion (MEX) is widely used commercially thanks to its affordability and recyclable materials, including filled filaments designed for enhanced thermal and mechanical properties [24,25,26]. However, the industrial applicability of these MEX polymers as insert materials in real IM tooling under production conditions has not been fully established, motivating the present study. Of all the materials that can be used in MEX, this research has focused on commercially available, polymer-based materials in filament form, which can be processed using standard equipment, excluding high-performance materials such as PEEK, PEI, and PEKK due to their cost and specialised processing requirements [27,28]. Some MEX filaments are marketed specifically for their high thermal resistance, mechanical strength, or thermal conductivity, often achieved through the addition of structural fillers. For example, Gohn et al. [2] successfully used neat PA6 (MatterHackers Pro Series Nylon, Lake Forest, CA, USA) and 12.5 wt% carbon fibre (CarbonX from 3DXTECH, Grand Rapids, MI, USA) to fabricate moulds for EVA injection. Similarly, Krizsma et al. [29] used 3D-printed Acrylonitrile Butadiene Styrene (Z-ABS from Zortrax S.A., Olsztyn, Poland) inserts to mould polypropylene (PP), producing 32 parts with 80% infill and 24 parts when infill was reduced to 25%. However, these studies did not provide detailed criteria for material selection.

A recent systematic review on additively manufactured inserts for injection moulding highlighted the growing interest in polymer-based tooling solutions, including MEX, while also emphasising the lack of standardised methodologies for material qualification and the limited experimental validation under real injection moulding conditions, particularly for commercially available filaments [21].

The same review reports that MEX-printed inserts have so far been tested mainly for a limited number of injection moulding cycles, typically ranging from a few units up to several tens of cycles, depending on the polymer system, infill strategy, and printing orientation. While these values are significantly lower than those reported for photopolymer-based or metal AM inserts, MEX remains attractive due to its low equipment cost, wide availability of thermoplastic filaments, short lead times, and suitability for rapid tooling and pre-series production. However, the literature consistently highlights critical limitations related to heat dissipation, dimensional stability, surface quality, and the lack of quantitative criteria for predicting insert durability under real processing conditions.

The current MEX polymer catalogue is dominated almost entirely by amorphous or low-crystallinity polymers such as poly(lactic acid) (PLA), acrylonitrile butadiene styrene (ABS), polycarbonate (PC), and glycol-modified polyethylene terephthalate (PETG). Semicrystalline polymers such as polypropylene (PP), polyamide (PA), and polyethylene terephthalate (PET) crystallise rapidly from the melt. This makes them more difficult to process, which is why they are less widespread [30]. Unfortunately, there is no information or in-depth studies on whether these materials can be used as inserts in injection moulding (IM) processes. For this reason, the aim of this article is to define which preliminary tests are suitable for qualifying a material as MEX-printable for inserts.

MEX filaments are generally available in different colours, either unfilled or containing functional/structural fillers that impart certain properties to the final object. Commercially available materials typically contain short glass, carbon, or Kevlar fibres to enhance mechanical properties, as well as metal particles or carbon-based fillers such as graphene to improve thermal conductivity. For each category of material, neat and filled solutions were selected. The main properties to take into account for the viability study were the mechanical stiffness in temperature, the thermal conductivity, and the CLTE. The extrapolated properties were used in a CAE analysis simulation using the commercially available software Moldex3D [31]. This allowed the temperature reached and the pressure resistance to be predicted. A screening of the possible admissible solutions was carried out by comparing the simulated conditions with the thermophysical characteristics. Selected filaments were then experimentally validated by 3D-printing inserts, assessing their dimensional accuracy, and testing them in real IM conditions. A steel insert with the same geometry was used to optimise the IM process before switching to AM inserts. All selected MEX materials successfully produced moulded parts, which were evaluated based on weight and showed no significant quality deviations.

A thermal imaging camera was employed to monitor insert temperatures during the IM cycle, especially at the point of part ejection, allowing a comparison with simulation predictions. It was thus possible to confirm the applicability of the materials planned for a small production and monitor its stability.

The methodology outlined in this study offers a generalised approach for evaluating MEX filaments for IM insert applications. By combining small-scale sample printing with relatively simple material characterisation and CAE simulations, the feasibility of a filament can be determined without the need for costly trial-and-error fabrication. This approach can also support the development of new materials tailored for specific IM challenges.

## 2. Materials and Methods

### 2.1. MEX Materials for Insert Production

Ten materials based on PLA, ABS, PA, PETG, and PET were considered. The complete list, along with the code used in the text, is shown in Table 1. Further details can be found in the Supporting Information.

### 2.2. Materials for Injection Moulding Process

Two materials with relatively low processing temperatures were selected: LDPE and PP.

The LDPE was purchased from Versalis (San Donato Milanese (MI), Italy) under the trade name Riblene MR 10 R and has the following main properties: density = 0.918 g/cm^3^, MFR = 20 g/10 min (190 °C, 2.16 kg), and a melting point of 107 °C.

The PP was purchased from LyondellBasell (Houston, TX, USA) under the trade name Moplen HP500N and has the following main properties: density = 0.90 g/cm^3^, MFR = 12 g/10 min (230 °C, 2.16 kg), and a melting point of 170 °C.

### 2.3. Characterisation Techniques

The rheological properties were evaluated using a TA Instruments ARES rheometer (TA Instruments, New Castle, DE, USA) with a strain-controlled parallel plate geometry (25 mm diameter). Preliminary strain sweep tests were carried out at ω = 10 rad/s and the lowest characteristic temperature to determine the linear viscoelastic region (LVR). Complex viscosity, storage, and loss moduli were measured during frequency scans from 10^2^ to 10^−1^ rad/s at different temperatures, with the strain amplitude set to ensure it fell within the LVR. Samples for rheological analysis (25 mm in diameter and 1 mm in thickness) were shaped using a Collin P 200 T hot plate press (COLLIN Lab & Pilot Solutions GmbH, Maitenberth, Germany) and held in a heated metal mould for two minutes at 100 bar.

The dynamic mechanical properties (DMA) were evaluated using a TA Instruments DMA Q800 with a single cantilever clamp in strain-controlled mode, and the same equipment was used for the CTLE test with a tension film setting. The DMA experimental conditions were set as follows: temperature range of 30–220 °C; heating rate of 3 °C/min; oscillation frequency of 1 Hz; and deformation amplitude of 0.05%. All tests were performed in accordance with ISO 6721 [32]. CTLE was estimated to be between 30 and 120 °C at a heating rate of 3 °C/min, with an applied preload of 0.001 N. The instrument measures the variation in sample length as a function of temperature, from which the CTLE between two chosen temperatures can be obtained, as described in ASTM E831 [33], ‘Standard Test Method for Linear Thermal Expansion of Solid Materials by Thermo-Mechanical Analysis’ [34].

Thermal conductivity measurements were performed using the Hot Disk^®^ TPS 2500 S instrument (Hot Disk AB, Gotebörg, Sweden), in accordance with the ISO 22007 standard [35]. This non-destructive analysis of thermal transport properties was conducted on two 20 × 20 × 10 mm^3^ square-based specimens. A 5465 Kapton sensor (Hot Disk AB, Gotebörg, Sweden) with a radius of 3.189 mm was placed between the two specimens, forming a kind of ‘sandwich’. The entire setup was placed in a thermostat bath at 23 °C for 10–20 min prior to commencing the measurement, to ensure a uniform temperature across all components. The heating power was set to 0.030 W, the measurement time to 160 s, the number of measurements to five scans, and the wait time between consecutive measurements to 300 s.

The filling percentage of the 3D-printed object was evaluated using the following formula:

(1) $$Filling\;percentage\;[\%]=\left(\frac{Weight\;specimen}{Volume\;specimen*Filament\;density}\right)\times 100\;$$

The weight of the specimens was measured directly from the 3D-printed objects, and the theoretical weight of the fully filled objects was calculated by multiplying the actual volume of the specimens by the filament density reported in the data sheets. All the dimensional measurements of the printed specimens that were necessary for the volume calculation were taken with a Mitutoyo Corp CD-15CPX digital calliper (Kawasaki, Japan) with an accuracy of 0.01 mm.

Specimen crystallinity was evaluated using differential scanning calorimetry (DSC). The DSC equipment used was a Q20 by TA Instruments, which required samples of 9 ± 1 mg. The chamber was purged with nitrogen at a rate of 50 mL/min. Each sample was heated from 0 °C to 200 °C or 300 °C at a rate of 10 °C/min in order to compare the crystallinity of the different 3D-printed objects. Cold crystallisation enthalpy (ΔHcc) and melting enthalpy (ΔHm) were evaluated as the integral of the peaks, as calculated using TA Instruments Universal Analysis 2000 software. The crystallinity of the artefacts is directly proportional to the difference between the melting and cold crystallisation enthalpies (ΔHm − ΔHcc).

During the injection moulding process, the Optris PI 640i infrared camera (Optris GmbH, Berlin, Germany) and Optris PIX Connect software Rel. 3.20.3111.0 were used to monitor the temperature profile of the mould and insert during the opening and ejection of the part, as well as during the additional post-cooling time added to the cycle to cool the insert.

Compression tests were performed in accordance with the ISO 604 standard [36] at room temperature using a Zwick-Roell Z100 dynamometer (ZwickRoell, Ulm, Germany) equipped with a 100 kN load cell, at a strain rate of 1 mm/min and with a preload stress of 0.1 MPa. Two specimens were used for each formulation: one for thermal conductivity tests on Hot Disk equipment and one for compression tests. The compression tests were carried out upon reaching a pre-established deformation of 2 mm. This limit was set to study the behaviour of the material within a good range of deformation (20% of the theoretical nominal size of the sample), as deformation beyond this point results in a loss of replication capacity and, thus, the production of objects of an unacceptable quality. The average values and corresponding standard deviations of maximum compression strength (s_max_), compression strength at 2 mm deformation (s_2mm_), deformation at 10MPa (ε _10MPa_), and deformation at 17MPa (ε _17MPa_) were determined.

### 2.4. Injection Moulding Object and Process Parameters

**Figure 1:**
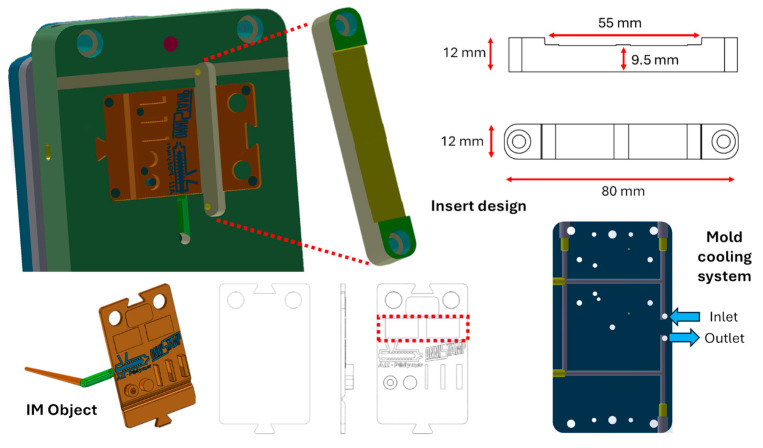
Geometry of the insert, injection-moulded object, and mould cooling system.

Figure 1 shows the geometry of a demonstrative plate produced by injection moulding. This object features the most common design elements found in commercial applications, including studs, holes, hinges, joints, different surface finishes, and logos. Furthermore, there is an insert (Figure 1) in a lateral part with respect to the gate, which can be replaced and produced with the desired material and process. This insert is relatively simple and small (80 × 12 × 12 mm^3^), making it suitable for the initial evaluation and validation of substitution feasibility.

The insert produces a surface area measuring 55 mm wide by 12 mm high with a thickness of 2.5 mm for the final object, accounting for approximately 7% of the object’s total surface area. Considering the volume of the IM object, the part in front of the insert accounts for approximately 14% of the total volume.

The insert was produced using traditional machining techniques in 1.2083 grade steel, while the MEX process was used with different commercial materials. The injection moulding process parameters were optimised using the steel insert to ensure good quality of the final object. Ten objects were moulded in sequence, using the steel insert as a baseline for comparison with objects produced using 3D-printed inserts. With all other parameters held constant, the packing pressure was increased progressively: to 10 bar in the first cycle, to 50 bar in the second, to 100 bar in the third, to 200 bar in the fourth, to 400 bar in the fifth, and to 600 bar in the sixth. For the remaining four shots, the pressure was maintained at 600 bar.

This transient packing pressure was chosen to avoid subjecting the inserts to maximum stress from the first shot, while also evaluating their possible progressive deterioration. The final five shots were selected to demonstrate the short-term feasibility of MEX inserts, rather than to assess long-term durability, which is beyond the scope of this study.

A shot size of 20 mm with an injection velocity of 20 cm^3^/s was adopted for all the shots to maintain a cushion in all moulding conditions. The commutation point between the injection and packing phases (also called the VP point) was set to occur when the part was filled to around 85% of the shot size (transfer position 5 mm from the end of the shot size), lasting 8 s to prevent shrinkage. The VP point is where machine control changes from flow rate control to pressure control. This commutation point is crucial in the process as it determines whether the maximum pressure will be high or low and can cause significant variations in pressure inside the cavity. Finally, a cooling time of 15 s was set prior to automatic mould opening and part ejection (total cycle time: 30 s). After 15 s, the moulded part reached the ejection temperature, at which point all of the material was solid and it was possible to extract the part without any problems. After ejecting the part, a residual cooling time of 60 s was applied between injection moulding cycles to further decrease the insert’s temperature and increase its lifetime. The thermal imaging camera recorded the temperature trend during the mould opening period for all cycles. At the end of the tenth cycle, a thermogram was recorded for two minutes.

The barrel temperature was set to 120 °C for LDPE and 180 °C for PP. The mould cooling system was set to 25 °C with a water flux of 10 L/min. The cooling channels are shown in Figure 1 and are positioned 24 mm below the mould closing surface. As the cooling system did not enter the insert part, the tool temperature was conditioned only by the mould surroundings. The injection moulding machine used was a Wittmann-Battenfeld SmartPower 50-130 (The WITTMANN Group, Kottingbrunn, Austria). All these parameters were used for the injection moulding simulations.

### 2.5. Simulation Software Data and Parameters

The IM simulation software used was Moldex3D Studio (version 2024R04). The geometry was drawn using CAD software and imported into the simulation software. The mould system was simplified in the software to include only the plates (300 × 200 × 160 mm^3^), in which the cavity was formed. The cooling system was extracted directly from the mould’s CAD model in Moldex3D, and the coolant flow was measured directly on the machine for accurate boundary conditions.

The mesh of the part and the insert were generated using Moldex3D’s Boundary Layer Mesh (BLM) technology, which automatically creates multiple prismatic boundary layers from the surface inward and fills the interior with tetrahedral elements. This approach enhances accuracy in capturing thermal and flow gradients near walls and interfaces. BLM settings such as mesh size, number of boundary layers, and offset ratios were tuned to balance accuracy and computational cost according to the model complexity. The mesh used for the cooling system was generated automatically with a hexa element in order to better describe the fluid behaviour related to the shape and to the path.

Approximately 900,000 solid elements were used: ~70,000 for the insert and ~230,000 for the part itself, with refined mesh density in regions of expected high gradients (gates, thin walls, and cooling surfaces) (see Supplementary Information for details).

Moldex3D allows the addition of new materials not present in its database. For each new material, relevant properties must be defined to ensure realistic simulations. To this end, the rheological behaviour of the moulding polymers was characterised near their melting temperatures, fitted using a modified Cross model, and the resulting parameters were input into the software (see Supporting Information for details).

Following direct material characterisation, the thermal conductivity and stiffness data for the inserts were entered into the software. Since both thermal capacity and conductivity vary with temperature, accounting for this variation is essential to improve the accuracy of the simulation and reduce the discrepancy between the simulation and real-world results.

The mould initial temperature was set to 25 °C, equal to the temperature of the water in the inlet point of the cooling channels; they also have fixed flow rate set in the process option and the air temperature was set to 23 °C. The polymer melt temperature at the inlet surface of the runner and process parameters were set to the values used for injection moulding.

Transient analysis was performed covering all steps of the injection cycle (filling, packing, and cooling). Moldex3D’s internal solver automatically manages the sequential coupling of these phases, using the end-state fields of one phase as initial conditions for the next. Default solver controls were adjusted to ensure stable temperature and pressure residuals throughout the simulation, with monitoring of convergence behaviour and step adjustments applied where necessary to prevent divergence or oscillatory solutions.

Simulations were run in Machine Mode, in which the specific injection moulding machine used in production was selected from the Moldex3D machine database and the real machine’s dynamic response and process settings (e.g., flow and pressure profiles) were applied directly in the simulation, ensuring that the virtual process closely reflected the actual equipment behaviour.

The numerical solver in Moldex3D employs a true-3D transient computational approach, solving the coupled flow and thermal and packing equations for the filling, packing, and cooling stages. Transient analysis allows us to start the simulation with an initial BC of the mould temperature equal to a single value (25 °C) and run multiple cycles to obtain a temperature distribution in the mould and insert different in any point and in each time step. Then, the software recalculates the distribution of the temperature starting with the new condition as the results of the previous cycle. The software control temperature distribution between every cycle and sets the correct cycle and status when the difference in the average mould temperature between the cycle is less than 1 °C or when the maximum cycle count was reached (set as 10 cycles).

This can ensure numerical stability and minimise oscillations in the temperature field. This approach allows accurate capture of fountain flow, shear heating, pressure evolution, and cooling dynamics across the part and mould.

### 2.6. MEX 3D-Printing Parameters

Several samples were 3D-printed using MEX technology to perform DMTA and Hot Disk analyses with a +45/−45° deposition infill pattern. Plate-shaped specimens measuring 35 × 10 × 2 mm^3^ were produced for the DMA analysis, and cubes measuring 20 × 20 × 10 mm^3^ were produced for the Hot Disk analysis. Once the materials had been selected, inserts in the shape shown in Figure 1 were produced with the same process parameters.

A Roboze One 3D printer (Bari, Italy) equipped with a 0.6 mm nozzle was used to prepare the specimens and inserts with the help of Simplify3D software 4.1.2 for slicing. All coils were dried in accordance with the manufacturers’ guidelines. The main variable printing parameters are summarised in Table 2, with a layer thickness of 0.2 mm, an infill percentage of 100%, and two outline shells maintained for all processes.

## 3. Results and Discussion

### 3.1. Characterisation of Selected Materials for MEX Insert Production

**Figure 2:**
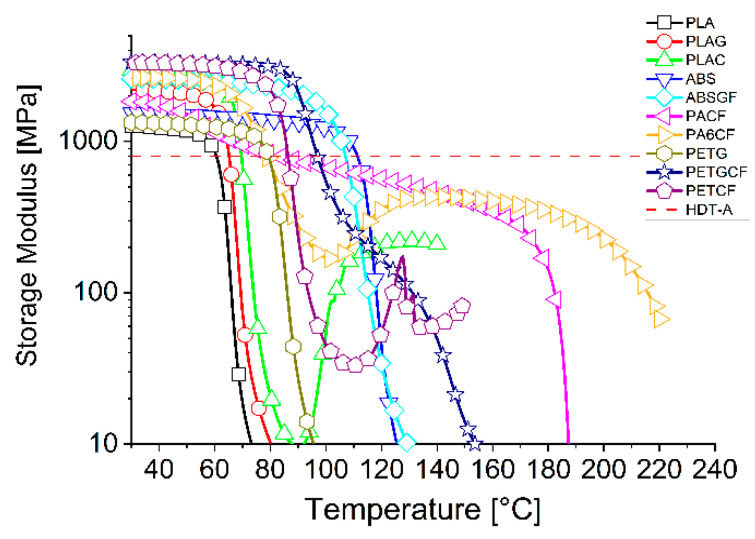
DMA storage modulus plots of MEX 3D-printed materials.

The first characterisation performed on the MEX-printed samples was DMA in cantilever mode with a temperature ramp, to evaluate variability in stiffness with temperature. The heat distortion temperature (HDT) at 1.82 MPa (HDT-A) was determined as the temperature at which the storage modulus reached 800 MPa, in accordance with the Takemori assumption [37]. The results of these analyses are summarised in Figure 2, which also reports the HDT-A value as a dotted line.

It should be noted that the PLA-based materials (PLA, PLAG, and PLAC) exhibit modulus decay above 60 °C, with an HDT-A value of 60–69 °C. Therefore, in terms of maximum usage temperature, there are minimal differences between unfilled and filled PLA.

ABS-based materials (ABS and ABSGF) exhibit a modulus drop similar to that of PLA, but at a higher temperature, with an HDT-A of 107–111 °C. Once again, the difference between the filled and unfilled materials is not significant.

Polyamide-based materials (PACF and PA6CF) exhibit a modulus flexion at slightly higher temperatures than PLA and have an HDT-A of 83 °C for PACF and 79 °C for PA6CF. Both filaments contain carbon fibres as a filler, according to the technical data sheets, with a higher quantity present in the PA6CF filament. The higher filler content does not appear to affect the temperature resistance of the samples. However, it should be noted that the manufacturers of PACF and PA6CF are different, so the performance of the matrices used may also differ and not be fully comparable. Furthermore, when observing the modulus curve after the initial property decay, PA shows an increase in modulus up to high temperatures, after which it decreases again above 200 °C. This behaviour is not evident in the PACF sample, which instead exhibits a much slower loss of properties. This difference could be due to the difference in matrix and filler mentioned previously. Under the adopted 3D-printing conditions, the PACF material is able to crystallise, whereas the PA6CF encounters more impediments, preventing organisation into crystals and resulting in a greater quantity of amorphous phase surviving. This portion of the material crystallises when the samples are heated during DMA testing (generally called ‘cold crystallisation’), resulting in an increase in the component’s temperature resistance. PETG-based materials (PETG and PETGCF) exhibit the initial modulus decay at temperatures higher than those of PLA, but similar to those of polyamides, resulting in an HDT-A of 79 °C for PETG and 97 °C for PETGCF. The presence of carbon fibre in the PETGCF sample significantly increases the first temperature drop compared to the neat matrix (+18 °C), and also results in a milder descent, highlighted by a flex around 120 °C.

Finally, PET-CF shows a similar trend to PETG, with a range of property loss around 86–90 °C. As with polyamides, PET-CF exhibits a recovery of properties at temperatures above 110 °C due to the ‘cold crystallisation’ behaviour of the amorphous portion.

Considering the results of the initial analysis, it is evident that heat treatment after production can increase the crystallisation of the material and therefore improve the artefacts’ thermo-mechanical resistance properties.

Two types of heat treatment were used: 80 °C for 60 min, labelled TT, and 140 °C for 60 min, labelled TT2. The selection of temperatures for the different materials was based on scientific research already published [38,39,40,41] and on observations of the curves in Figure 2. The temperatures must be higher than the Tg of the material, at which point a loss of mechanical properties begins to occur (see Figure 2), but not so high as to render the material extremely deformable and unable to maintain its expected shape.

**Figure 3:**
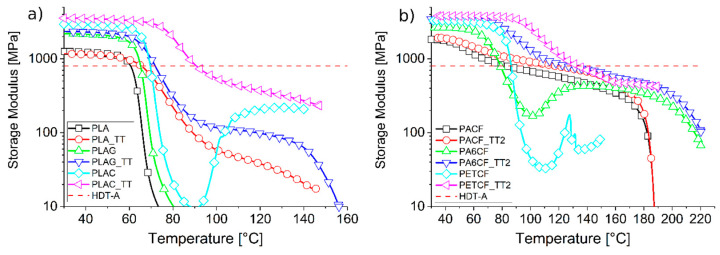
Comparisons of DMA storage modulus plots of MEX 3D-printed materials based on PLA (**a**) with or without thermal treatment TT and based on PA and PET (**b**) with or without thermal treatment TT2.

Figure 3 shows the main results obtained by comparing 3D-printed materials with and without heat treatment. The graphs show that PLA-based materials benefit greatly from TT. PLA increases by approximately 5 °C in HDT-A, while PLAG increases by 6 °C. The best result is achieved by the PLAC material, which increases by 20 °C in HDT-A.

The PACF material achieved an impressive increase of 34 °C with the TT2 treatment, while the second polyamide considered (PA6CF) exhibited a significant enhancement in HDT-A, rising from 79 °C to 126 °C. This result was predictable based on the recrystallisation behaviour observed during the initial heating in the previous DMA analysis.

Similarly, the PETCF sample exhibited clear cold crystallisation; indeed, the heat-treated sample exhibited a similar increase in HDT-A, rising from 86 °C to 133 °C.

ABS- and PETG-based materials were also considered; however, they do not benefit from heat treatment as they do not crystallise.

These behaviours can be explained by the presence of a crystalline component in such samples. When DSC analyses are compared for the untreated samples and those treated with TT or TT2, the enthalpy of melting (ΔHm) is substantially higher than that of crystallisation upon heating (ΔHcc) in the PLA, PA, and PET-based samples. Conversely, PACF has a very high crystallinity even without heat treatment (ΔHm − ΔHcc = 38.1 J/g), so the effect of TT2 is marginal in terms of both the crystalline content and stiffness at different temperatures (see Table 3). ABS and PETG essentially have no crystalline fraction, so heat treatment does not provide any benefits [39].

**Figure 4:**
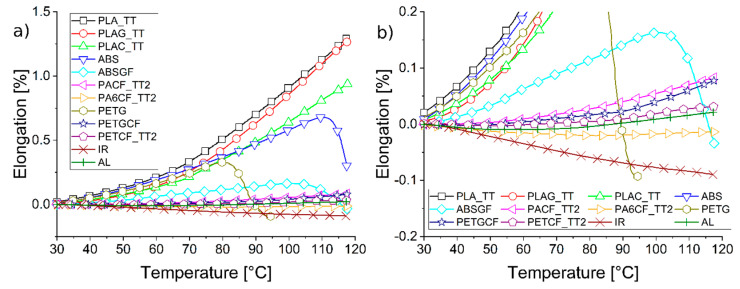
Linear thermal expansion plot from 30 °C to 120 °C of MEX 3D-printed samples. (**a**) whole graph; (**b**) zoom at small Elongation %.

The second property of the materials analysed using DMA in tensile mode is linear thermal expansion. The samples were subjected to a heating ramp and their dimensional variation was evaluated as a percentage compared to the initial dimensions (see Figure 4 for elongation vs. temperature). Since the material of the instrument clamps also undergoes thermal expansion, the graphs were used to compare the materials, reporting the thermal expansions recorded by steel (IR) and aluminium (AL).

As the effectiveness of the heat treatments for thermo-mechanical resistance with the PLA, PA, and PET-based formulations had been verified, thermal expansion analyses were carried out after such treatments. Steel has the minimum elongation as a function of temperature, which is why it is the lowest curve in Figure 4. The elongation of an aluminium alloy from which moulds and mould inserts can be produced has also been reported. As is well known, the CTLE of steel (e.g., 1.2083 grade 10.5 10^−6^ K^−1^) is lower than that of aluminium (e.g., 7075 grade 23.6 10^−6^ K^−1^), which is why the aluminium curve is above the iron curve in the graph. PA6CF_TT is the only material tested that falls between the steel and aluminium curves. Consequently, this material is most similar to the metals taken into consideration. However, PETCF_TT also appears to behave similarly to AL, so this solution can also certainly be taken into account, as can PETGCF and PACF, which have only slightly higher elongation than AL. The PLA base materials (PLA, PLAG, and PLAC) demonstrate far greater consistency in elongation at different temperatures than ABS and PETG.

Finally, the presence of glass fibres in ABSGF reduces the elongation due to thermal expansion compared to neat ABS, but it is still significantly higher than materials based on PA and PET.

Another important property that a material for inserts needs to have is the ability to dissipate heat. For this reason, specific MEX 3D-printed samples were characterised using Hot Disk analysis, and the results are reported in Table 4. The same samples were also weighed and measured for dimensions in order to obtain the filling percentage, which is also reported in Table 4.

Neat polymers such as PLA, ABS, and PETG have the lowest thermal conductivity coefficients, with expected values around 0.2 W/m⸱K. Adding graphene to PLA (PLAG) improves its performance without making drastic changes, as does adding glass fibres to ABS. In all these cases, the density of the printed objects is around 96%.

Conversely, adding carbon fibres to all formulations has a positive impact on thermal conductivity, which increases drastically in all cases studied. PETG and PET reach 0.37–0.43 W/(m·K) (PETGCF and PETCF, respectively), while polyamides have a conductivity of 0.42–0.55 W/(m·K) (PACF and PA6CF, respectively), which is approximately double that of neat polymers. The density of samples containing carbon fibres is slightly lower than previously reported, except for PETCF. This means that these objects will have more empty space inside them, which could result in lower thermal conductivity and thermo-mechanical stress resistance.

Finally, the PLAC sample has a conductivity similar to solutions containing carbon fibres at 0.46 W/m⸱K, thanks to the great thermal conductivity of copper metal particles of around 400 W/m⸱K.

As a general comparison, it should be noted that the metallic materials used to produce the moulds have thermal conductivities that are clearly higher than those measured here. Steel X40Cr14, also coded 1.2083, has a conductivity of 20 W/m⸱K, and aluminium 7075 has a conductivity of 130 W/m⸱K, according to the manufacturers’ technical data sheets.

Finally, the mechanical resistance of the insert materials must be evaluated. Compression tests were carried out on the 20 × 20 × 10 mm^3^ samples used for thermal conductivity testing. Table 5 shows the maximum allowable stresses and deformation at specific loads.

The PLA_TT, PLAG_TT, and PETG materials exhibit maximum load peaks within a deformation range of up to 2 mm, whereas all the other materials demonstrate an increasing isotropic behaviour without any detected maximums within this range (see Supplementary Information). PLAG_TT and PA6CF_TT2 exhibit the highest loads at approximately 100 MPa, whereas ABS, PACF_TT2, and PETG demonstrate the lowest, with maximum stress occurring at approximately 60 MPa.

At the end of this material characterisation campaign, significant differences among the investigated 3D-printed solutions clearly emerge. However, these differences alone do not allow one to assess whether a given material is suitable for the specific injection moulding application. For this reason, an injection moulding process simulation is required to provide the critical thermo-mechanical information needed for reliable material screening.

### 3.2. Simulation of IM Process with Insert

The Moldex 3D simulation software, when filled with the parameters of the materials used and the injection moulding conditions, simulated the process and provided important results regarding the applicability of the materials as inserts. In particular, the pressures acting on the insert’s surface with different injected materials and their temperatures were taken into consideration.

The simulations carried out concerned the interaction between the two main process variables, namely the material injected into the cavity and the insert material. These two variables are reported in the first two columns of Table 6. All process parameters were kept constant and are reported in Section 2.

Simulations were carried out with the materials with the best and worst thermal conductivity to check for the greatest variations in conditions in contact with the insert. The worst-performing insert material is PLA, while the best is PA6CF. Two injected materials were chosen: LDPE and PP. For both solutions, the performance of an insert made of steel was also compared.

Of all the results that the software could provide, we focused on predicting the temperature and pressure in the insert area.

We focused on the prediction of temperatures and pressures in the insert area, among all the results that the software can provide.

The temperature data refers to three different stages of the process: the end of the injection phase (EOF), when the cavity has been completely filled with molten material; the end of the cooling step (EOC), when the mould is opened; and after the sample has been extracted (Open). The injection phase generally lasts for fractions of a second (0.7 s in this case), while the holding and cooling steps are longer (8 + 15 s), as is the extraction (3.7 s in this case).

The maximum temperature is only marginally useful as it is localised to a single point on the insert surface. By moving a few millimetres from the maximum point and enlarging the area under investigation, the temperature decreases by several degrees (see Supplementary Information Figures S4–S8). For this reason, the average temperature and standard deviation of the exposed surface are reported in Table 6 (Avg T EOF ± SD).

During the initial injection phase, there is a sudden increase in temperature, particularly on the surface, involving a few tenths of a millimetre of the insert thickness. The simulation software also allows a temperature probe to be positioned under the surface in order to monitor the process and detect any problems with the material used (see Supplementary Information Figure S8). A point 0.5 mm deep below the maximum surface heating point was taken as an example and coded as ‘probe’ (see Table 6 for the ‘Max T EOF probe’ data).

Table 6 also reports the maximum pressure value reached in the insert area during the entire process (Max P). All simulations use a packing pressure of 600 bar on the sprue.

The first criterion adopted to assess material suitability concerns the temperature reached by the insert during the moulding cycle. Simulation-predicted temperatures were compared with those obtained from DMA measurements, specifically the HDT-A values. A material was classified as unreasonable if its HDT-A was more than 10 °C below the simulated temperature range, risky if it fell within ±10 °C of the simulated range, and reasonable if its HDT-A exceeded the simulated temperature by at least 10 °C. Since the temperature distribution within the insert is non-uniform, two representative values were considered for the comparison: the average temperature (Avg T) and the maximum temperature recorded by the probe (see Supplementary Information Tables S2 and S3, Figure S8). Based on this first screening, PLA_TT was excluded from LDPE moulding, while PLAG_TT and PETG were classified as risky. For PP moulding, only PACF_TT2, PA6CF_TT2, and PETCF_TT2 were classified as reasonable, whereas ABS, ABSGF, and PETCF_TT2 showed a limited safety margin and PLAC_TT presented a higher thermal risk.

The second screening criterion was related to the pressure applied to the insert surface during mould filling and packing. Simulations showed that LDPE, due to its higher viscosity at the selected processing temperature, generated higher cavity pressures than PP, with values of approximately 16–17 MPa for LDPE and around 10 MPa for PP (Table 6). To assess mechanical admissibility, compression test results were used. From the stress–strain curves, deformation values corresponding to stresses of 17 MPa and 10 MPa were extracted and compared (Table 5). Since no universally defined deformation limit exists, as admissibility strongly depends on the specific application and insert geometry, deformation thresholds were interpreted in a relative manner. In this study, displacement values on the order of 0.2 mm were considered acceptable, whereas values exceeding approximately 0.4 mm were regarded as excessive and potentially detrimental to dimensional accuracy.

For LDPE moulding, where the applied pressure is higher, the deformation at 17 MPa was considered. Although PLAG_TT and PETG were thermally classified as risky, both exhibited among the lowest deformation values and were therefore retained for subsequent insert production.

For PP moulding, deformation values at 10 MPa were evaluated. Among the thermally admissible materials, PACF_TT2 and PA6CF_TT2 exhibited the highest absolute deformation and were therefore considered critical; between the two, PA6CF_TT2 showed a more favourable overall balance and was retained, while PETCF_TT2, despite relatively high deformation, was also maintained due to its superior HDT-A. Conversely, PETGCF, classified as thermally risky and showing the highest deformation, was discarded. ABS, ABSGF, and PLAC_TT, although limited by temperature resistance, displayed low deformation and were therefore retained for experimental validation.

Overall, this combined thermo-mechanical screening resulted in nine admissible material solutions for LDPE and five for PP. Consequently, only the insert–polymer combinations classified as reasonable were manufactured and further investigated experimentally.

In conclusion, semicrystalline polymers, such as PA- and PET-based filaments, consistently outperformed amorphous materials, particularly when reinforced, due to their superior thermo-mechanical stability and dimensional retention under cyclic moulding conditions. Among all materials, the semicrystalline PA6 filament with the highest carbon fibre content (PA6CF) exhibited the most robust behaviour, combining high stiffness at processing temperatures with enhanced thermal conductivity, resulting in minimal thermal accumulation, limited dimensional deviation, and the shortest additional cooling times. Filled materials generally performed better than neat polymers, with highly reinforced systems providing the best performance, whereas filaments containing low amounts of conductive fillers, such as graphene, did not show significant improvements under the tested conditions. Amorphous polymers, while less resistant to high temperatures, remain suitable for short-run or low-stress applications where high performance is not critical.

### 3.3. Insert Characterisation

**Figure 5:**
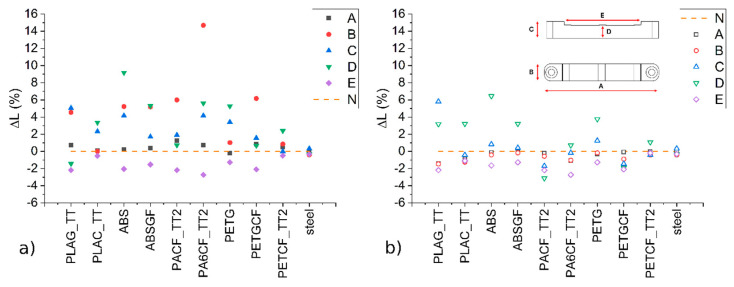
Dimensional deviations in A, B, C, D, and E points compared to the nominal set values (ΔL = (L measured − L nominal)/L nominal * 100). Plot (**a**) refers to insets as printed/plot and (**b**) as after post-processing smoothing.

The first evaluation of the MEX 3D-printed inserts is their dimensional accuracy. Since they must be coupled with other metal parts of the mould, their suitability is essential. All inserts were measured at five points labelled A to E, as shown in Figure 5. Using the adopted MEX production conditions, some dimensional deviations were observed compared to the nominal set values in the CAD file, and these differences are reported as a percentage in Figure 5 (ΔL = (L measured − L nominal)/L nominal * 100). The nominal size is marked with a dotted line corresponding to 0 on the same graph and labelled N; thus, anything above this line is oversized. The steel insert was also measured and added to the graph for comparison.

During the MEX 3D-printing production, the inserts almost always have material that extends beyond the perimeter limit, which is why they are larger and thicker than expected. This can be seen from the positive deviation in dimensions A, B, C, and D, while dimension E is an internal part of the insert design and is therefore almost always undersized (Figure 5a). Based on these findings, inserts that were too large were manually smoothed using abrasive paper to achieve the desired dimensions. Post-processing consisted of manual sanding using abrasive paper to remove excess material from the external surfaces until the nominal dimensions were reached, requiring only simple tools and limited operator skill.

The two dimensions that are most important for fitting to the mould are A and B; therefore, PLAG_TT, ABS, ABSGF, PACF_TT2, PA6CF_TT2, and PETGCF require more work. Conversely, the closer the dimensions are to the nominal ones, the better they should be considered. In this case, the PLAC_TT and PETCF_TT2 materials practically did not require any post-processing, as small deviations in the C dimension (i.e., height) can be compressed by the IM press during the first cycle, and the D dimension does not render the insert unusable but rather results in a smaller cavity.

After post-processing, all the inserts have slight variations compared to the reference, but they can still fit the metal mould hole (Figure 5b).

To obtain inserts quickly and economically, the best solutions are therefore PLAC_TT and PETCF_TT2, as they do not require post-processing, whereas most of the other materials require manual dimensional correction, increasing lead time and production cost. Thanks to this characterisation, it is also possible to predict the percentage of undersizing of designs exported to 3D printers and to anticipate the need for post-processing, thereby enabling a more objective evaluation of insert manufacturability and reducing or even eliminating finishing operations.

### 3.4. IM Part Production and Characterisation

**Figure 6:**
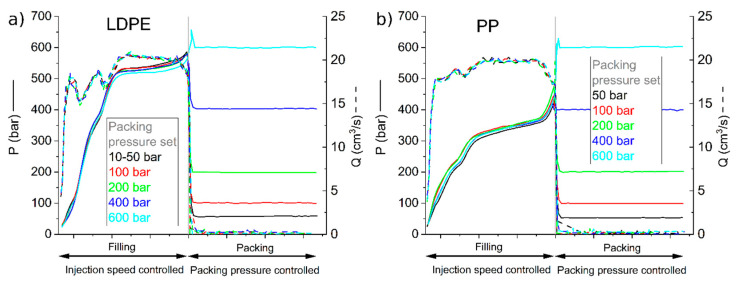
Cavity pressure graph given by injection moulding press software for LDPE (**a**) and PP (**b**).

As defined in Section 2, the moulding process was optimised using a steel insert, and the same conditions were adopted for the other inserts. To adopt a progressive stress process and identify any issues, the holding pressure was increased from 10 to 600 bar during the first six cycles. The IM machine can measure the pressure applied to the material using a sensor in the final part of the barrel, as well as the position of the screw inside the barrel, during the process. Using these two pieces of data, the machine produces a graph showing the range of pressure (P) and material flow (Q) during the IM printing cycles, as shown in Figure 6.

The simulation software predictions reported in Table 6 can be confirmed experimentally from the two plots, since pressures of approximately 550 bar are reached for LDPE and 350 bar for PP in the filling process.

Notes on the object production phase are reported below, as they may be important for subsequent considerations. Due to the geometry of the insert, major problems in the injection moulding process arise from the infiltration of molten material into the spaces between the insert and the mould, as well as into the screw seats that keep the insert anchored to the mould. If such leaks are significant, they do not allow for the correct extraction of the object through the ejection pins, meaning that the object remains stuck to the insert. In this case, removal must be carried out manually. This problem almost always occurred with the PACF_TT2 insert and also occurred in two cases with the PETGCF insert. These two materials exhibited the greatest deformation under the moulding conditions (Table 5), making material infiltration possible. In all other cases, infiltration did not prevent the IM object from being extracted from the mould.

Videos were acquired with a thermal camera during all the production cycles and analysed with the Optris PIX Connect software. Analysing the video makes it possible to export a graph showing the temperature trend in the mould insert area once the object has been expelled.

All graphs show the same trend: an initial peak as soon as the mould is opened, followed by a progressive decrease in temperature over time. The maximum temperature and the rate at which it decreases are directly influenced by the thermal conductivity of the materials used and the adhesion between the insert and the mould.

**Figure 7:**
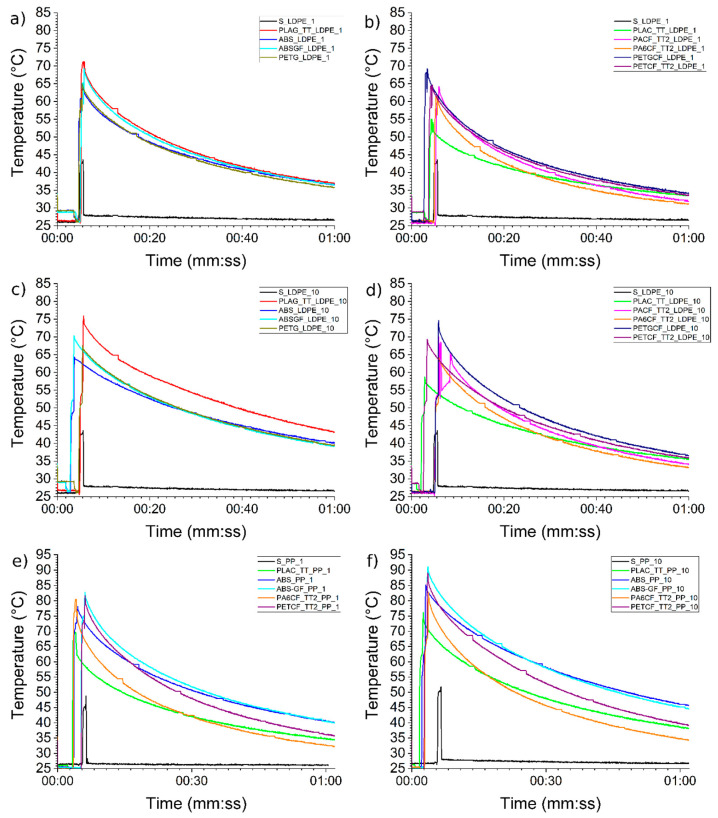
Graph of temperature registered by thermo-camera after the first cycle with LDPE (**a**,**b**) and PP (**e**); at the end of the tenth cycle with LDPE (**c**,**d**) and PP (**f**).

For each insert, the maximum temperatures during the first and last cycles were obtained and compared with those of a steel part of the mould located far from the insert, in order to evaluate the temperature deviations. Figure 7 plots such temperatures collected by conductivity groups and IM materials: materials with conductivity close to 0.2 W/m⸱K (a and c), materials with conductivity close to 0.5 W/m⸱K (b and d), materials used for LDPE production (e), and materials used for PP production (f).

Figure 7a shows the temperature recorded in the first moulding cycle. The temperature peak is around 71 °C for PLA, 70 °C for ABS-GF, and 65 °C for PETG and ABS. The simulation predicted a maximum temperature of 70 °C, lowered to 63 °C at the end of the opening and extraction process (see Supporting Information), and an average temperature of 61 ± 8 °C for printing a low-conductive material such as PLA. The experimental results closely match these predictions (see Table 6).

The second set of materials plotted in Figure 7b refers to the materials with the highest conductivity. The PETGCF material had the highest registered temperature of 69 °C and was also the material with the lowest conductivity among those containing carbon fibres (Table 4). PACF_TT2 and PETCF_TT2 show maximum temperatures of 64 °C and 62 °C, respectively. Surprisingly, the sample with the best performance is PLAC_TT, with a maximum temperature of only 55 °C. Considering the simulations performed, all materials except PLAC_TT performed as expected (65 °C EOC and 57 °C open; see Supporting Information).

The slight temperature difference compared to the simulation could be due to poor adhesion between the insert and the mould. This results in a lack of heat dissipation from the insert to the surrounding metal, causing a higher temperature. The good performance of the PLAC_TT insert could be due to the CTLE being higher than that of the metal (see Figure 4 and Figure 5, and Table 4), which gives it a greater propensity to adhere to the mould surface and fill any possible gaps.

Finally, the temperature reached at the end of the additional one-minute cooling period between the first and second moulding cycles must be considered. For the less conductive inserts, the temperature difference between the insert and the mould is between 11 and 7 °C (Figure 7a) and between 7 and 4 °C for the more conductive ones (Figure 7b). This fact will generate a progressive increase in the temperature in the subsequent moulding cycles [9]. For this reason, the graphs of the temperature at the end of the 10th injection moulding cycle are also shown in Figure 7c,d. As expected, the failure to return to the starting temperature in the first cycle resulted in a slight increase in the maximum temperatures reached by all inserts, at both the peak and the end of the cooling period. The PACF_TT2 sample (+7 °C) and the PLAG_TT sample (+6 °C) showed the largest increases in temperature, while the PA6CF_TT2 sample (+2 °C) showed the smallest.

As previously mentioned, the PACF_TT2 sample had some extraction difficulties and therefore had greater contact time with the insert than the other samples, resulting in a higher temperature at the end of the 10th moulding cycle. The PA6CF_TT2 and PLAG_TT samples’ best and worst performances are instead predictable from their measured thermal conductivity (Table 4).

The gradual temperature increase during subsequent IM cycles also increases the time required to reach the final temperature registered at the end of the first IM cycle. The additional cooling time, quantified in overtime with respect to the 60 s, is generally between 13 and 30 s for the PA6CF_TT2 and PLAG_TT, respectively.

The production cycle time with a steel insert is 30 s, which must be increased by 60 s for 10 cycles, and a further 13 s at the end of the tenth cycle with the best MEX insert (PA6CF_TT2). Therefore, it is possible to produce approximately 39 objects per hour with the best MEX insert, which is much less than the 120 obtainable with the metal insert. This solution is therefore unacceptable for mass production, but it can be adopted for niche productions of small quantities, where productivity is less important than the ability to vary the solution.

The same procedure was followed for producing PP-based objects. The inserts used for PP are the same as those already used with LDPE; thus, the inserts have already undergone the stresses of the first ten moulding cycles.

As expected, given the higher melt temperature of PP compared to LDPE, the maximum temperatures reached by the inserts are higher. In particular, the simulation predicted temperature ranges of 81 ± 13 °C for high-conductive inserts and 88 ± 15 °C for low-conductive inserts (see Table 6).

The thermal imaging camera recorded a temperature of 80 °C in the first cycle, increasing to 83 °C in the tenth for PA6CF_TT2 (see Figure 7e,f). Therefore, for the PA6CF_TT2 insert, the real application’s performance is very close to that predicted by the simulation.

The small temperature increase in only 3 °C between cycles required a further 13 s of cooling to return to the conditions of the first cycle. The theoretical productivity is therefore the same as that previously reported for LDPE.

In a published solution, epoxy-based resin was used for the inserts, alongside IM PLA or PP [9]. An additional 260 s of cooling were used in a 217 s cycle that already included 180 s of cooling. The authors of this study also demonstrated how using conformal cooling in such inserts increases dissipation efficiency by 70%, while greatly reducing the additional time required. Therefore, the study reported here presents similar time extensions, which could be reduced by the possible use of conformal cooling.

The PETCF_TT2 insert shows lower performance than the PA6CF_TT2 insert, which is entirely predictable given the lower thermal conductivity coefficient of the material (see Table 4). Indeed, the insert is warmer in the first production cycle (+1 °C) and in the tenth (+6 °C) than the PA6CF_TT2 solution. This increases the time needed to return the initial cycle conditions to 21 s compared to 13 s for the PA6CF_TT2 solution.

The PLAC_TT solution presents the lowest temperature in the first cycle (70 °C), but the highest increase of about +6 °C during the ten cycles. This is due to the lower conductivity coefficient of this material, meaning that the accumulated thermal energy takes longer to be transported outside. The extra time calculated for this solution is 26 s.

For the two inserts with low thermal conductivity (ABS and ABSGF), the thermal imaging camera detected maximum temperatures of 78 and 83 °C, which increased to 85 and 91 °C, respectively, in the tenth cycle. The higher temperatures and greater increases during the cycles are due to the lower thermal conductivity coefficient. In this case too, the temperatures predicted by the simulation (88 ± 15 °C) are in line with those measured. The additional times calculated for these solutions are 19 and 26 s, respectively.

From a quality perspective, objects printed with 3D-printed inserts have a different surface finish to those with metal inserts. A visual assessment is provided in the Supplementary Information. Although no insert visual failure was observed within the ten moulding cycles, the expected life-limiting mechanisms for MEX inserts include time-dependent fatigue under pressure, progressive surface wear due to melt flow, and permanent deformation of thin or highly stressed regions.

**Figure 8:**
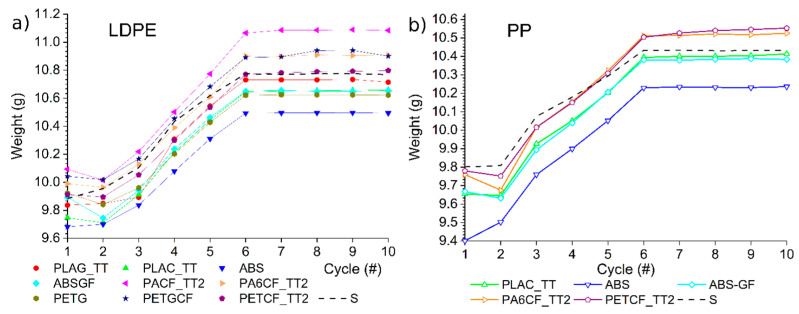
Sample weight from cycle 1 to 10 for the production of LDPE (**a**) and PP (**b**) objects with different inserts.

To quantitatively evaluate the quality of the injection-moulded (IM) objects, with particular attention to permanent deformation phenomena, all samples were weighed. The part weight was used as an indirect indicator of cavity filling and dimensional stability, as it is expected to increase during the first six cycles due to the progressive increase in packing pressure, and then stabilise during the final four cycles (cycles 6–10), when identical processing conditions are applied. By comparing the weight of samples moulded under the same conditions with those produced using the steel insert (Figure 8), it is possible to assess the influence of the insert material and its deformation on the quality and consistency of the IM parts.

A weight lower than the reference value can indicate a larger insert, resulting in a smaller cavity to fill. A decrease in weight during moulding may mean that the insert increases in volume during the process, leaving less space for the product. Conversely, an increase in weight may indicate collapse of the insert or removal of material during cycles (wear) that generate a greater free injection volume.

The PLAG_TT, PLAC_TT, ABS, and ABSGF PETG inserts always produce LDPE samples that are lighter than the reference (Figure 8a). Conversely, the PACF_TT2, PA6CF_TT2 and PETGCF inserts are heavier. The ABS case has the lowest weight of all those analysed in Figure 8a, at around 3% less than the others in all cycles. This is because the insert has smaller cavity dimensions, as shown in Figure 5 by the D and E data, and therefore, the weight of the sample is also lower. Conversely, the PACF_TT2 always has a higher weight in Figure 8a, exceeding 3% in the final cycles. In this case too, the reason lies in the dimensional qualities of the produced insert. The D and E dimensions of the PACF_TT2 insert in Figure 5 are all lower than the nominal dimensions, meaning the cavity and the weight of the produced sample are larger.

The PA6CF_TT2 insert produces objects with an LDPE weight most similar to the steel reference (dotted line) in the first five cycles, while the PETCF_TT2 insert produces objects with an LDPE weight most similar to the steel reference in the last five cycles (Figure 8a). These two inserts also have dimensions that are closest to the nominal data (Figure 5). It is therefore evident that the dimensional quality of the insert is the main factor affecting the quality of the final moulded object.

The less rigid materials should undergo the largest deformations during the injection phases. Of the materials that showed higher deformation under load (PACF_TT2, PA6CF_TT2, PETGCF, and PETCF_TT2, as shown in Table 5, all tend to be above the steel comparison curve because they compress more, creating a larger cavity.

In any case, an assessment of weight stability was carried out by calculating the weight variation in the IM samples in the last five cycles (standard deviation/mean value * 100). Using the steel insert results in a variation of 0.03% for LDPE and 0.02% for PP. A threshold value of 0.06% was set for weight variation to report any anomaly in IM parts with MEX-based inserts.

In the last five IM cycles shown in Figure 8a, the weight of the sample could generally be considered constant (with a variation of less than 0.06%); therefore, there were no evident variations in the dimensions of the insert that would cause an increase in weight. Some insert solutions are just above the threshold: 0.07% for PLAG_TT, probably due to the material’s low resistance at temperature, as highlighted by the HDT results (Table 4); and 0.09% for PACF_TT2, due to the insert’s low mechanical properties, which are already evident at room temperature (Table 5). The only case of noteworthy variation (0.23%) is the PETGCF insert, where the low mechanical properties are compounded by the numerous manual extractions of the parts, which can cause further damage to the insert surface and, thus, variation in weight.

With regard to the IM of PP, the PLAC_TT insert produced a weight trend similar to that obtained with LDPE, but closer to the reference obtained with the steel insert. The samples appear to have a minimal tendency to increase in weight during the last five cycles (0.07% variation), which is a great result for a material with relatively low temperature resistance (HDT at 91 °C), but with one of the lowest deformations for the assumed pressures in the IM (0.23 mm).

As in the case of LDPE production, the PA6CF_TT2 exhibits behaviour that is slightly closer to the reference weight in the first five IM cycles at low holding pressures, and slightly heavier but consistent between them in the last five cycles (0.05% variation). This material was one of those with the highest deformations under load, so this variation in weight is understandable.

The object obtained with the PETCF_TT2 insert exhibits behaviour that is almost identical to that of the PA6CF_TT2. The only difference is that the latter shows a tendency to increase in weight in the final IM cycles (0.18% variation), which could indicate a problem with the insert increasing the volume of the cavity. There is no obvious explanation for this, since the material has good temperature resistance (HDT-A), but it has a great distribution of deformation under compressive stress, which could mean the 3D-printed part performs non-uniformly.

As expected from the previous IM with LDPE, the ABS and ABSGF inserts produce pieces with a lower weight than the reference. Both materials demonstrate optimal stability over the final five cycles, with a mere 0.03% weight variation. This excellent result is due to the compression performance achieved (Table 5) and the fact that, although the HDT-A was considered at risk because it was very close to the value predicted by the simulations, it was still sufficient.

## 4. Conclusions

This paper aims to investigate the applicability of additive manufacturing (AM) technology for producing mould inserts for injection moulding (IM), specifically through the material extrusion (MEX) process using fused filament fabrication. This approach enables the manufacturing of limited-edition parts and quick design changes to injection-moulded components.

The thermal and mechanical properties of the materials were investigated through the rapid and cost-effective production of samples using 3D-printing equipment. Dynamic mechanical thermal analysis (DMTA), linear thermal expansion, thermal conductivity measurements, and compression strength tests were performed to screen potentially suitable materials.

Injection moulding simulations were conducted using Moldex3D software. The software was used to assess the thermal and mechanical stresses experienced by the insert during moulding by inputting material properties, process parameters, mould geometries, and boundary conditions.

Based on the simulation results, nine of the ten tested materials were deemed suitable for use under LDPE moulding conditions and five for PP. These nine commercial filaments were then used to manufacture inserts, the dimensional accuracy of which was evaluated. Six of the inserts required significant post-processing to fit the mould cavity; however, all of the materials were successfully used in further applicability testing.

Injection moulding trials were carried out using two commercial thermoplastics. To progressively increase the thermal and mechanical stress on the inserts, the production process began with LDPE, which was then switched for PP due to its higher melting point.

All inserts retained their structural integrity throughout ten injection moulding cycles, consistently producing undistorted parts. A cooling interval of just over one minute was sufficient to prevent insert overheating between cycles. Monitoring with an infrared thermal imaging camera revealed a gradual and continuous increase in temperature at the insert surface over the ten cycles. The PA6CF_TT2 insert performed best, requiring only 13 s of additional cooling in the tenth cycle to return to initial conditions. This was expected, given that the material had the highest thermal conductivity of those tested.

Among the investigated materials, the semicrystalline PA6 filament reinforced with the highest carbon fibre content (PA6CF) exhibited the best performances.

Conversely, the PLAC_TT sample exhibited the lowest temperature increase during the first cycle thanks to its good thermal conductivity and excellent mould adhesion, which is due to its higher thermal expansion relative to steel—again, confirming the initial material characterisations.

These results highlight that, regardless of whether a material is amorphous or semicrystalline, and irrespective of filler content, CAE simulations are essential to predict the thermo-mechanical performance of each candidate filament under actual injection moulding conditions, allowing for informed material selection before committing to physical insert production.

All materials predicted to be suitable passed the practical injection moulding tests, and the temperatures recorded by the thermo-camera aligned with those expected in the simulations.

The dimensional accuracy of the 3D-printed inserts was reflected directly in the moulded products, and remained consistent across repeated prints for most materials, with weight variation of less than 0.06%. Only a few materials (e.g., PLAG_TT, PACF_TT2 and PETCF_TT2 for LDPE, and PETCF_TT2 for PP) exceeded this threshold due to lower mechanical strength or thermal resistance. ABS and ABSGF demonstrated excellent stability, albeit with less accurate dimensions. Therefore, if deviations from the nominal dimensions occur, geometric compensation must be considered during the design of the insert.

Future research should explore the use of composite filaments with tailored filler content to further enhance thermal conductivity and stiffness and the long-term durability of inserts under extended IM cycles. Additionally, integrating real-time thermal feedback into the CAE simulations could further improve predictive accuracy for industrial-scale applications.

In addition, the flexibility of additive manufacturing enables the functional variation in insert geometry: future developments may include the integration of surface embossed text or logos, non-planar profiles that modify part thickness or that introduce hinges or even a barrier wall to intentionally interrupt melt flow. Such strategies could enable functional tuning of injection-moulded parts without redesigning the entire mould.

In conclusion, the preliminary tests used in this study were decisive and sufficient for identifying suitable materials for insert production. The results demonstrate that MEX-based AM using filament feedstock is a viable alternative method for producing injection moulding inserts for small production batches, offering reduced costs and shorter lead times.

## Figures and Tables

**Table 1:** List of MEX materials used and codes.

Base Materials	Filler Type and Amount from Manufacturer Datasheets	Trade Name	Code
PLA	-	Water Blue PLA from Geeetech (Shenzhen, China)	PLA
PLA	Graphene Plus (unknown amount)	Grafylon 3D from Filoalfa (Torino, Italy)	PLAG
PLA	60 wt% copper particles	HTPLA from Protopasta (Champaign, IL, USA)	PLAC
ABS	-	Performance ABS filament from TreeD Filaments (Seregno, Italy)	ABS
ABS	25 wt% glass fibres	ABS-GF25 Filament from QIDI TECH (Shenzhen, China)	ABSGF
PA	carbon fibres (unknown amount)	Alfanylon CF from Filoalfa (Torino, Italy)	PACF
PA 6	15 wt% carbon fibres	PA Carbon Pro from Roboze (Bari, Italy)	PA6CF
PET-G	-	PETG Orange from Filalab (Calenzano, Italy)	PETG
PET-G	10 wt% carbon fibres	Alfaomnia from Filoalfa (Torino, Italy)	PETGCF
PET	15 wt% carbon fibres	Raise3D Industrial PET CF (Irvine, CA USA)	PETCF

All materials were dried following the recommendations on the manufacturers’ technical data sheets.

**Table 2:** MEX 3D-printing process parameters.

Material	Extrusion Temperature [°C]	Extrusion Speed [mm/s]	Extrusion Width [%]	Outline Overlap [%]	Bed Temperature [°C]	Fan Speed [%]
PLA	220	30	130	50	50	50
PLAG	220	50	130	50	40	50
PLAC	195	20	130	50	60	0
ABS	230	40	130	50	80	0
ABSGF	270	40	130	50	110	0
PACF	250	50	100	100	70	0
PA6CF	250	50	100	100	70	60
PETG	250	40	130	50	80	0
PETGCF	250	50	100	100	70	0
PETCF	290	40	130	50	70	50

All eventual heat treatments were carried out on MEX 3D-printed objects in a ventilated oven produced by Memmert (Schwabach, Germany).

**Table 3:** DSC data from the first heating cycle on MEX 3D-printed samples without or with heat treatments (enthalpy of cold crystallisation ΔHcc; enthalpy of melting ΔHm).

Sample	ΔHcc [J/g]	ΔHm [J/g]	ΔHm − ΔHcc [J/g]
PLA	23.9	30.0	6.1
PLA_TT	-	23.5	23.5
PLAG	23.6	25.0	1.4
PLAG_TT	4.0	20.0	16.0
PLAC	9.5	15.2	5.7
PLAC_TT	-	16.1	16.1
PACF	1.9	40.0	38.1
PACF_TT2	-	45.6	45.6
PA6CF	10.7	30.7	20.0
PA6CF_TT2	-	3.5–36.6	40.1
PETCF	13.7	13.6	-
PETCF_TT2	-	34.1	34.1

**Table 4:** Thermal conductivity, filling percentage, and HDT-A and qualitative thermal expansion evaluation of the MEX 3D-printed materials.

Material	Thermal Conductivity [W/m⸱K]	Filling Percentage [%]	HDT-A [°C]	Thermal Expansion
PLA_TT	0.22 ± 0.01	96.4 ± 0.5	65	-
PLAG_TT	0.25 ± 0.01	96.8 ± 0.3	72	-
PLAC_TT	0.46 ± 0.01	97.1 ± 0.4	91	-
ABS	0.19 ± 0.01	95.1 ± 0.6	111	-
ABSGF	0.22 ± 0.01	97.5 ± 0.1	107	+
PACF_TT2	0.42 ± 0.01	81.0 ± 1.1	118	++
PA6CF_TT2	0.55 ± 0.01	83.4 ± 2.2	126	+++
PETG	0.23 ± 0.01	96.8 ± 0.2	79	-
PETGCF	0.37 ± 0.01	83.1 ± 3.5	97	++
PETCF_TT2	0.43 ± 0.01	95.4 ± 0.4	134	++

- insufficient; + sufficient; ++ good; +++ excellent.

**Table 5:** Compression tests main data of MEX 3D-printed inserts (average values and corresponding standard deviations of compression strength when a deformation of 2 mm is reached (s_2mm_), maximum compression strength (s_max_), deformation at 10 MPa of compression strength (ε_10MPa_), and deformation at 17 MPa of compression strength (ε_17MPa_) were reported.

Material	s_2mm_ [MPa]	s_max_ [MPa]	ε_10MPa_ [mm]	ε_17MPa_ [mm]
PLA_TT	71.20 ± 0.97	76.99 ± 1.14	0.46 ± 0.03	0.58 ± 0.04
PLAG_TT	98.12 ± 0.60	102.67 ± 1.33	0.23 ± 0.03	0.33 ± 0.03
PLAC_TT	73.29 ± 0.78	73.29 ± 0.78	0.23 ± 0.02	0.31 ± 0.02
ABS	58.13 ± 0.99	58.13 ± 0.99	0.18 ± 0.01	0.27 ± 0.01
ABSGF	78.62 ± 1.17	78.62 ± 1.17	0.18 ± 0.03	0.27 ± 0.03
PACF_TT2	60.3 ± 0.8	60.3 ± 0.8	0.42 ± 0.01	0.57 ± 0.01
PA6CF_TT2	95.2 ± 1.1	95.2 ± 1.1	0.41 ± 0.03	0.55 ± 0.03
PETG	57.86 ± 0.16	62.70 ± 1.14	0.19 ± 0.01	0.29 ± 0.01
PETGCF	69.0 ± 3.1	69.0 ± 3.1	0.32 ± 0.02	0.47 ± 0.03
PETCF_TT2	84.59 ± 1.98	84.59 ± 1.98	0.27 ± 0.12	0.37 ± 0.14

**Table 6:** Moldex 3D simulation data at the end of filling (EOF) and end of cooling (EOC).

Injected Material	Insert Material	Avg T EOF ± SD (°C)	Max T EOF Probe (°C)	Max P (MPa)	Avg T EOC ± SD (°C)
LDPE	PLA_TT	94 ± 7	81	16	61 ± 8
LDPE	PA6CF_TT2	92 ± 6	80	17	57 ± 7
LDPE	Steel	39 ± 1	38	17	33 ± 1
PP	PLA_TT	128 ± 13	108	10	88 ± 15
PP	PA6CF_TT2	124 ± 12	104	10	81 ± 13
PP	Steel	53 ± 1	49	10	40 ± 1

## Data Availability

The original contributions presented in this study are included in the article/Supplementary Materials. Further inquiries can be directed to the corresponding author.

## Supplementary Materials

The following supporting information can be downloaded at: https://www.mdpi.com/article/10.3390/ma19020369/s1, Additional data on materials, Simulation software data and parameters, Characterisation of selected materials for MEX insert production, Simulation of IM process with insert and Videos acquired with thermos-camera. Figure S1. Rheological curves of LDPE and PP used for injection moulding; Figure S2. Mesh of mould, cooling channels, insert and part in Moldex3D software. Figure S3. Stress–deformation curves of all the materials used in compression mode; Figure S4. Insert temperature prediction at the end of filling step (EOF) from Moldex3D simulation software with insert made of PLA, PA6CF and steel and injection moulding PP (upper plots) and LDPE (lower plots); Figure S5. Insert temperature prediction at the end of filling step (EOF) from Moldex3D simulation software with insert made of PLA, PA6CF and steel and injection moulding PP (upper plots) and LDPE (lower plots). The insert is sectioned in correspondence with the maximum surface temperature (reported in the label; Figure S6. Insert temperature prediction at the end of cooling step (EOC) from Moldex3D simulation software with insert made of PLA, PA6CF and steel and injection moulding PP (upper plots) and LDPE (lower plots); Figure S7. Insert pressure prediction at the end of filling step (EOF) from Moldex3D simulation software with insert made of PLA, PA6CF and steel and injection moulding PP (upper plots) and LDPE (lower plots); Figure S8. Insert temperature prediction during the entire process from Moldex3D simulation software with insert made of PLA, PA6CF and steel and injection moulding PP and LDPE. Probe 1 is on the surface corresponding to the maximum temperature point and Probe 2 is under the Probe 1 at a depth of 0.5 mm. The dotted lines correspond to the HDT-A temperatures obtained with the various materials; Figure S9. Thermo-camera pictures of objects before ejection from the mould; Figure S10. Thermo-camera pictures of inserts after ejection; Figure S11. Visual comparisons of the parts of the LDPE injection-moulded objects that correspond to the inserts; Table S1. Calculated parameters put in Moldex3D software as Modified Cross Model; Table S2. Moldex 3D simulation data at the end of filling (EOF), end of cooling (EOC) and after opening and eject (Open); Table S3. Insert temperature prediction from Moldex3D simulation software compared to HDT-A.
