# Supplementary material for: Validation of Filament Materials for Injection Moulding 3D-Printed Inserts Using Temperature and Cavity Pressure Simulations
Source: Materials (Basel). 2026 Jan 16;19(2):369. doi: 10.3390/ma19020369 (PMC12842723; doi:10.3390/ma19020369)
Supplement: Supplementary file 1 [file materials-19-00369-s001.zip › materials-4088601-supplementary.pdf]

Validation of Filament Materials for Injection Moulding 3D-Printed Inserts Using Temperature and Cavity Pressure Simulations

Daniele Battegazzore [1], Alex Anghilieri [2], Giorgio Nava [2] and Alberto Frache [1,*]

[1] Dipartimento di Scienza Applicata e Tecnologia, Politecnico di Torino, Alessandria Site, V.le Teresa Michel 5, 15121 Alessandria, Italy; daniele.battegazzore@polito.it
[2] Moldex3D Italia, Larco Caleotto 30, 23900 Lecco, Italy; alexanghilieri@moldex3d.it (A.A.); giorgionava@moldex3d.it (G.N.)
* Correspondence: alberto.frache@polito.it

SUPPORTING INFORMATION

MEX materials for insert production

Water Blue PLA from Geeetech is a neat PLA filament (coded as PLA). Chamber temperature = 25-60°C; Extrusion temperature = 185-220°C; Tensile Strength: ≥ 60Mpa.

Grafylon 3D from Filoalfa is a PLA reinforced with Graphene Plus from Directa Plus (coded as G). Chamber temperature = 0-50°C; Extrusion temperature = 170-230°C; Printing speed = 40-120 mm/sec; Thermal conductivity = 0.385 W/mK.

Copper-filled Metal Composite HTPLA from Protopasta is a metal PLA filament that can be heat-treated to firm up parts and hold shape up to near melting point at 175°C (coded as COP). Chamber temperature = 60°C; Extrusion temperature = 195°C; Printing speed = 20-30 mm/sec.

Performance ABS filament from TreeD Filaments is a neat Acrylonitrile Butadiene Styrene filament with excellent shine and tactile feel (coded as ABS). Chamber temperature = 100°C; Extrusion temperature = 230°C; Printing speed = 60-90 mm/sec; Tensile modulus 2300 MPa; Vicat softening temperature 90°C.

ABS-GF25 Filament from QIDI TECH is a glass fiber-reinforced ABS. It has a co-extrusion 'skin-core' structure: the outer 'skin' of the filament is a modified resin with high layer adhesion, and the inner core is reinforced resin containing high chopped fiber content. The co-extrusion skin-core technology has greatly increased fiber content while maintaining the toughness of the filament and thus improved the mechanical properties as well as heat resistance of printed parts (coded as ABSGF). Chamber temperature = 100-110°C; Extrusion temperature = 250-270°C; Printing speed = 30-120 mm/sec; Tensile strength 48 MPa; Young's modulus 3750 MPa; Heat deflection temperature method A 81°C.

Alfanylon CF was purchased from Filoalfa, it is a PA reinforced with carbon fibers (coded as AN). Chamber temperature = 60-70°C; Extrusion temperature = 250-270 °C; Printing speed = 30-50 mm/sec. Tensile strength 125 MPa, Young's modulus 8.9 GPa, Elongation at break 4.9 %, HDT 163°C.

PA Carbon Pro was purchased from Roboze, it is a PA 6 reinforced with carbon fibers (15% by weight) (coded as PA). Chamber temperature = 90 °C; Extrusion temperature = 270 °C; Printing speed = 3600 mm/min; Layer height = 0.27 mm; Infill percentage = 100%. Tensile strength at 25 °C in XY orientation at ±45° 141 MPa, Young's modulus 8.8 GPa, Elongation at break 2%. Glass transition temperature (Tg) 70°C, melting temperature (Tm) 234°C, and temperature of crystallization (Tc) 180°C.

PETG Orange was purchased from Filalab, is a neat PET-G filament (coded as PETG). Chamber temperature = 75-90°C; Extrusion temperature = 240-260°C; Printing speed = 50-100 mm/sec. Tensile strength 50 MPa, Flexural modulus 2.1 GPa, HDT 64°C.

Alfaomnia was purchased from Filoalfa; it is a PET-G + 10% carbon fiber + additives (coded as AO). Chamber temperature = 0-70°C; Extrusion temperature = 240-260 °C; Printing speed = 40-60 mm/sec. Tensile strength 111 MPa, Young's modulus 7.74 GPa, Elongation at break 3.2 %, HDT 109°C.

Raise3D Industrial PET CF (coded as PETCF) is a 15wt % carbon fiber-reinforced composite filament based on PET (Polyethylene terephthalate). After annealing, PET CF stabilizes its heat resistance at around 150ºC and tensile modulus and strength of over 6 GPa and 80 MPa, respectively. Chamber temperature = 60-80°C; Extrusion temperature = 280-300°C; Printing speed = 35-90 mm/sec.; Tensile strength 87 MPa, Bending strength 123 MPa, HDT 112°C.

Simulation software data and parameters

Moldex 3D simulation software is able to add materials with respect to those present in the database to be closer to reality. The obtained rheological curves (Figure S1) were then fitted using a modified Cross model (Equations (S1)–(S4)) to calculate the parameters (Table S1) to be put in the Moldex3D software. The state equation that related P, volume and temperature was also modeled inside the software with its specific model, taking the raw data from the measurements.

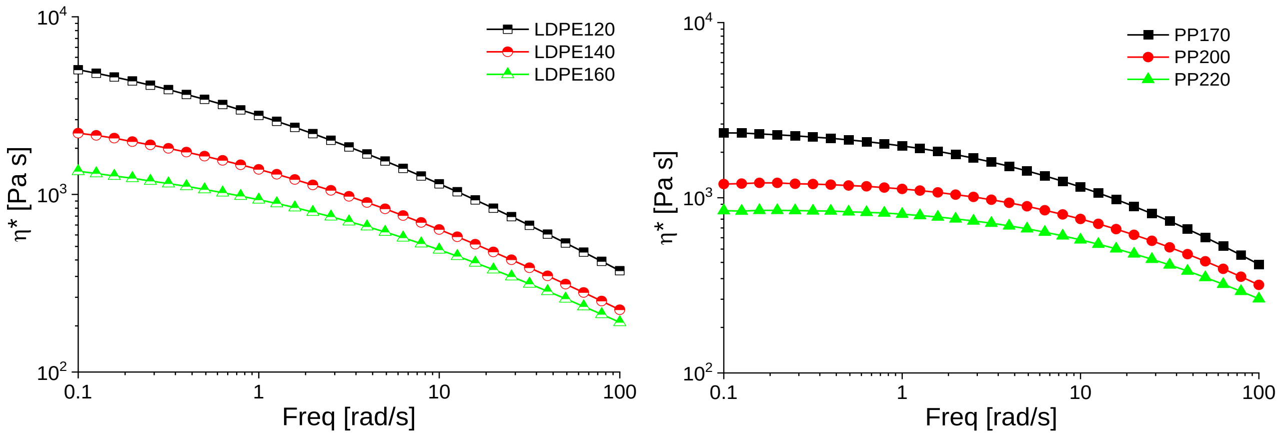

Figure S1. Rheological curves of LDPE and PP used for injection molding.

$$\eta = \frac{\eta_0}{1+\left(\frac{\eta_0\dot{\gamma}}{\tau^*}\right)^{1-n}} \quad \text{(S1)}$$

$$\eta_0 = D_1 \cdot e^{\left(\frac{-A_1(T-T_c)}{A_2+(T-T_c)}\right)} \quad \text{(S2)}$$

$$T_c = D_2 + D_3P \quad \text{(S3)}$$

$$A_2 = \widehat{A_2} + D_3P \quad \text{(S4)}$$

Table S1. Calculated parameters put in Moldex3D software as Modified Cross Model.

Material	n	τ^* [Pa]	D1 [Pa s]	D2 [K]	D3 [K/Pa]	A1	$\widehat{A_2}$ [K]
LDPE	0.4734	3060.7	3.537*1017	223.15	0	41.175	51.6
PP	0.3489	19.735	5.307*109	263.15	0	19.018	51.6

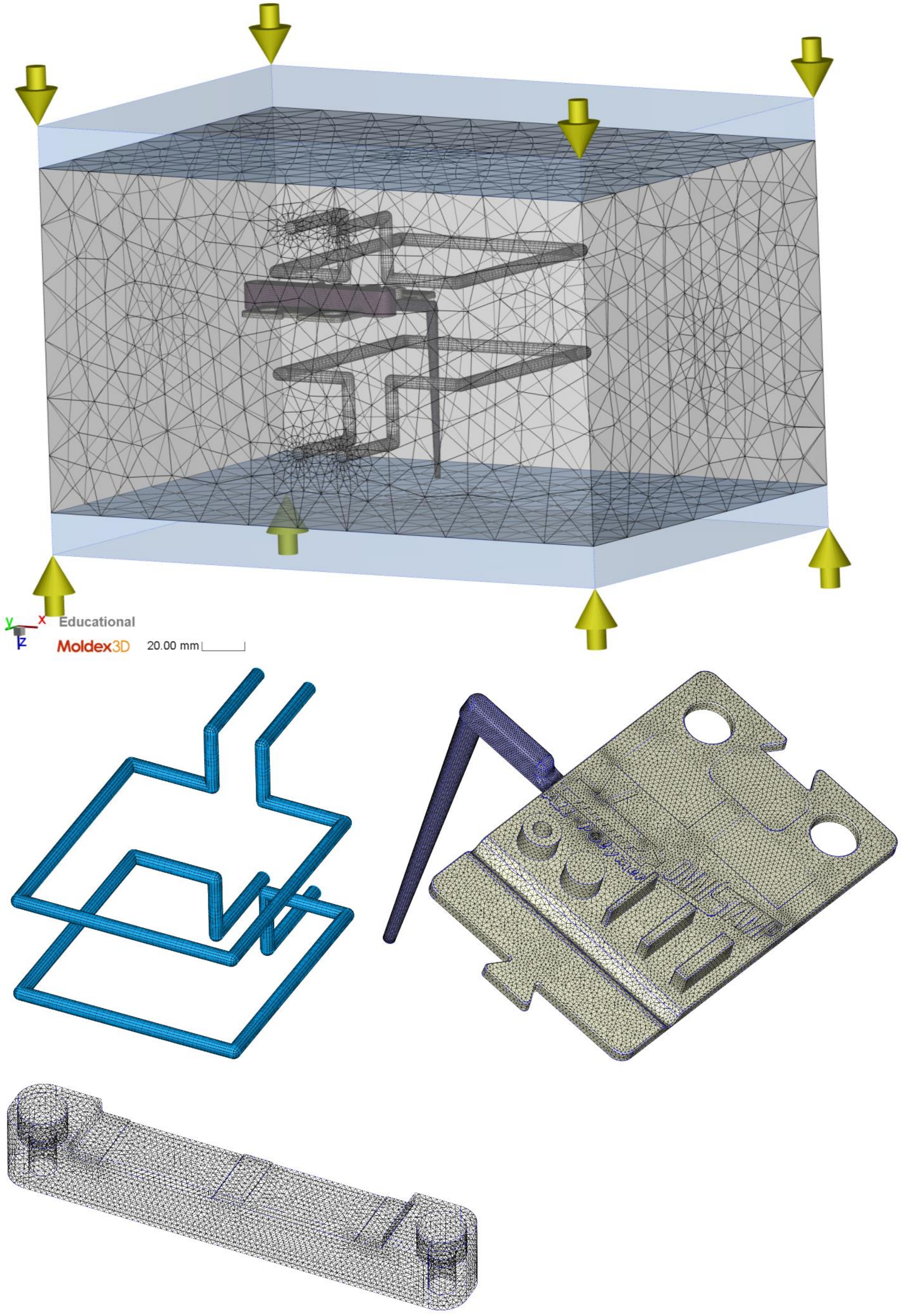

Figure S2. Mesh of mold, cooling channels, insert and part in Moldex3D software.

Characterization of selected materials for MEX insert production

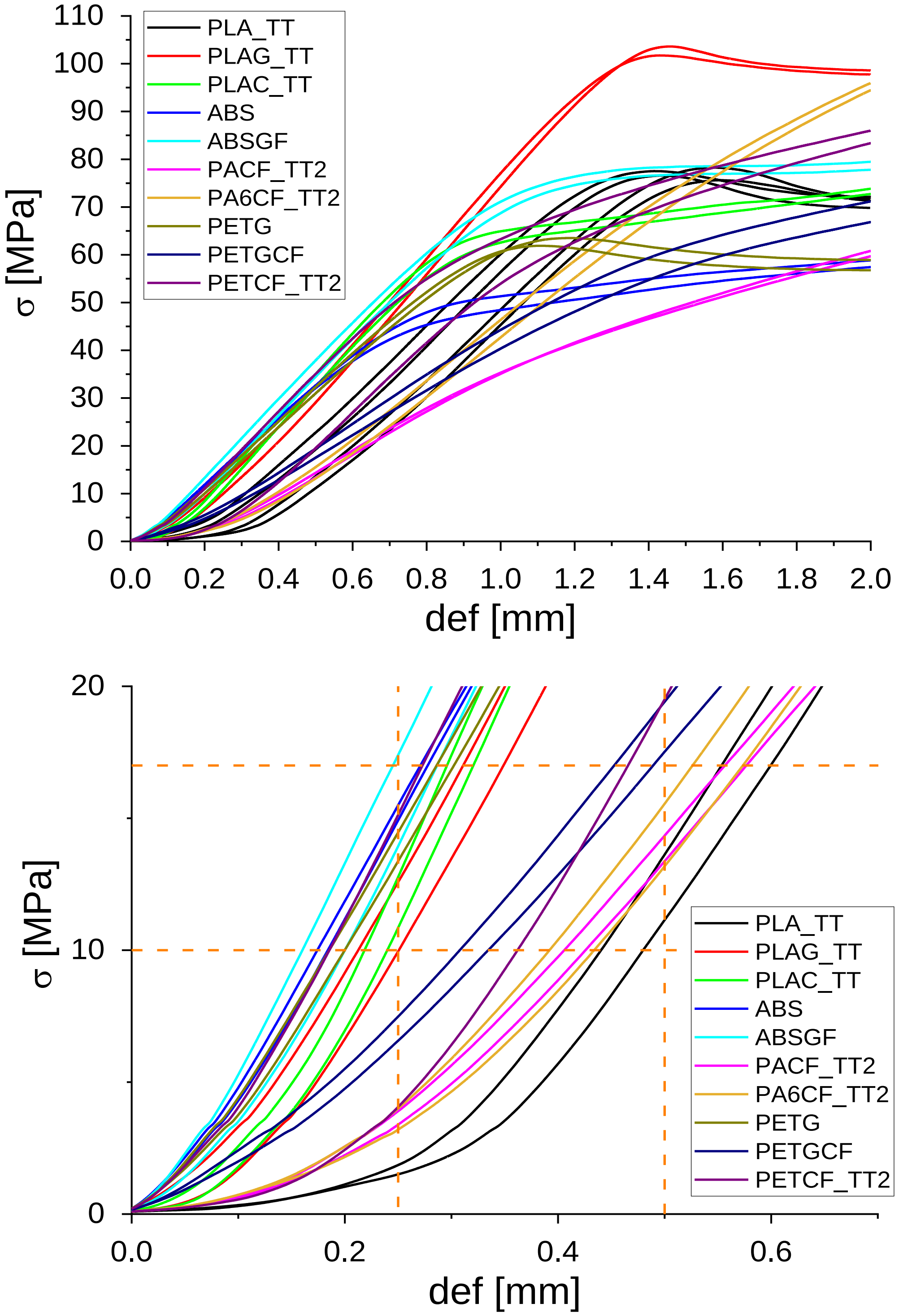

Figure S3. Stress-deformation curves of all the materials used in compression mode.

Simulation of IM process with insert

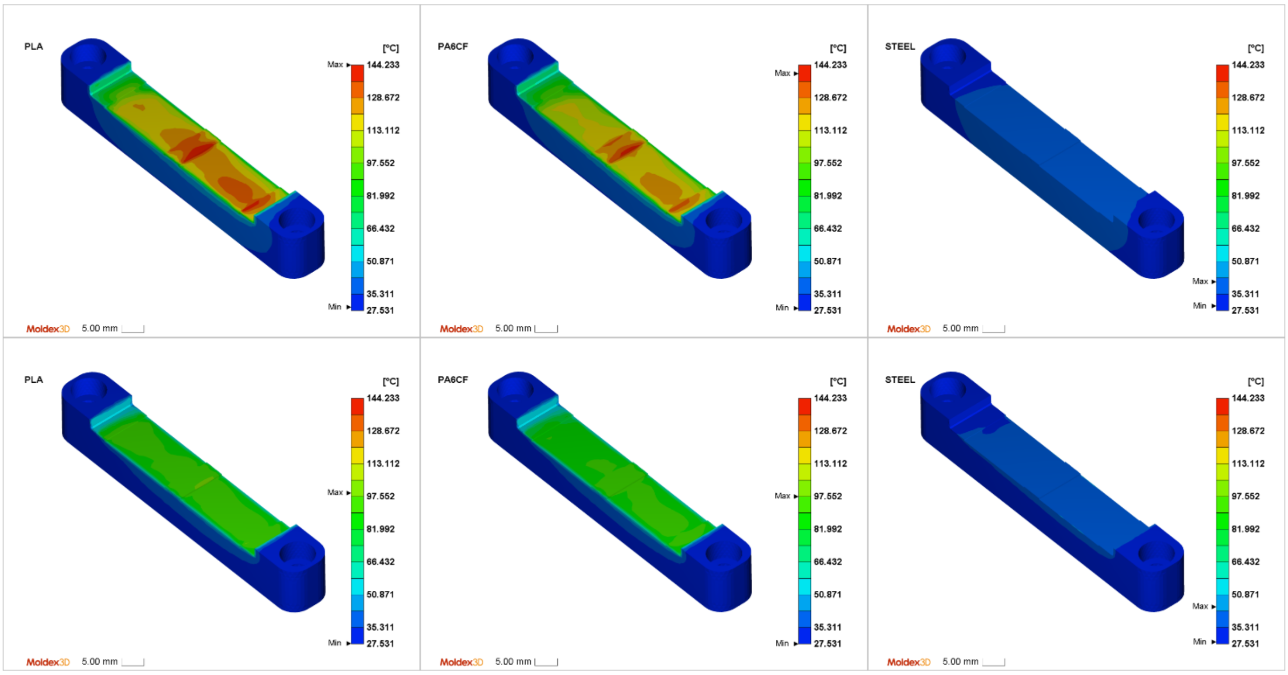

Figure S4. Insert temperature prediction at the end of filling step (EOF) from Moldex3D simulation software with insert made of PLA, PA6CF, and steel and injection molding PP (upper plots) and LDPE (lower plots).

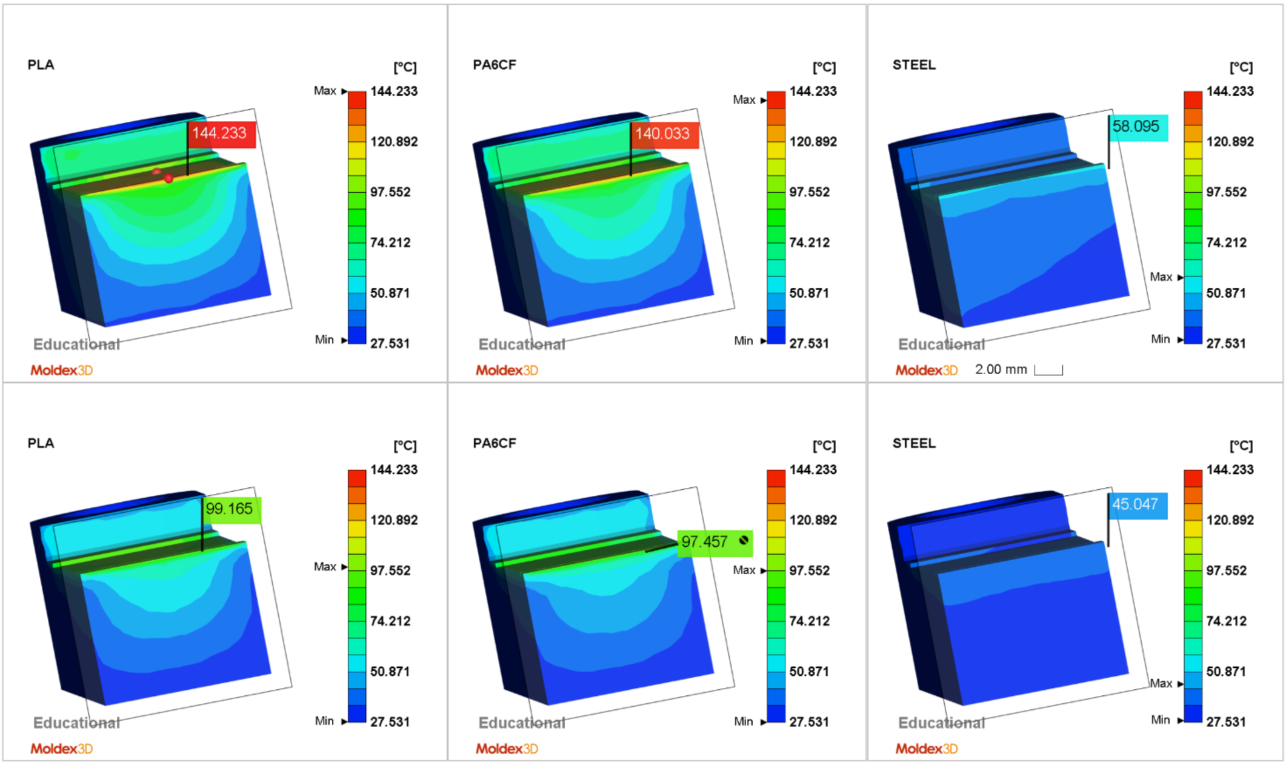

Figure S5. Insert temperature prediction at the end of filling step (EOF) from Moldex3D simulation software with insert made of PLA, PA6CF, and steel and injection molding PP (upper plots) and LDPE (lower plots). The insert is sectioned in correspondence with the maximum surface temperature (reported in the label).

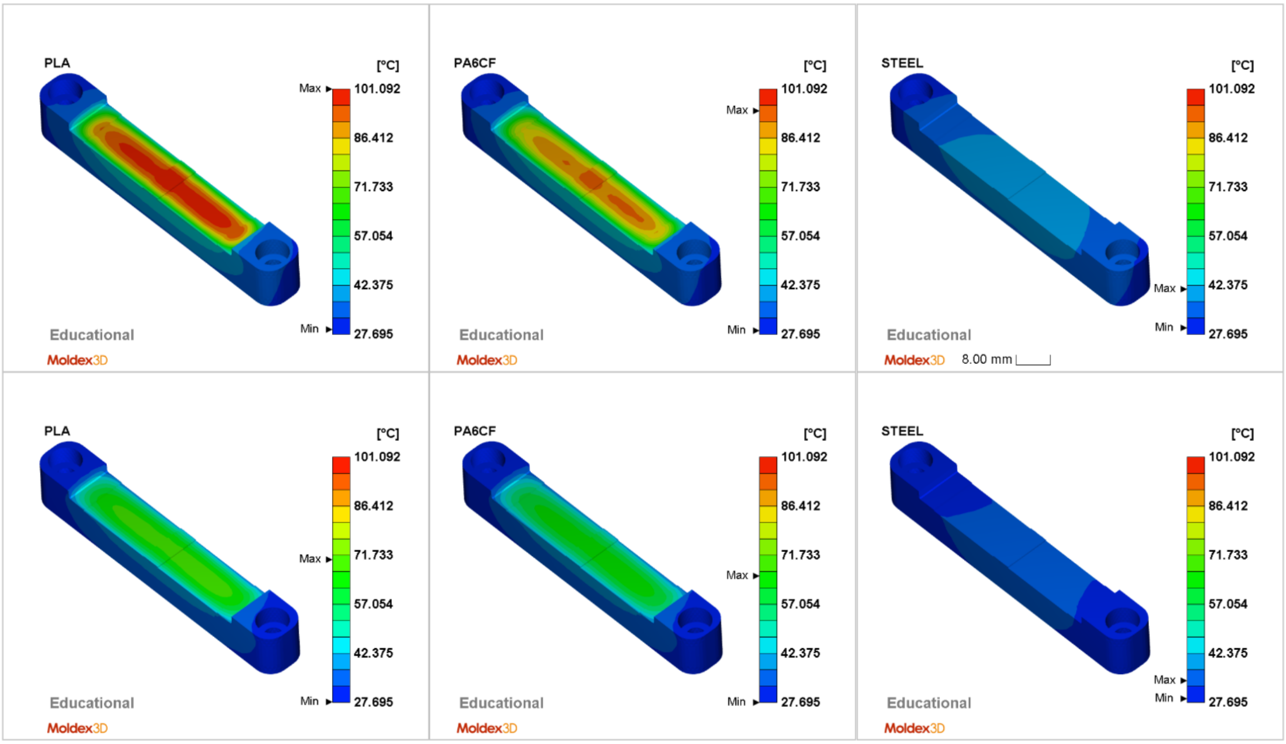

Figure S6. Insert temperature prediction at the end of cooling step (EOC) from Moldex3D simulation software with insert made of PLA, PA6CF, and steel and injection molding PP (upper plots) and LDPE (lower plots).

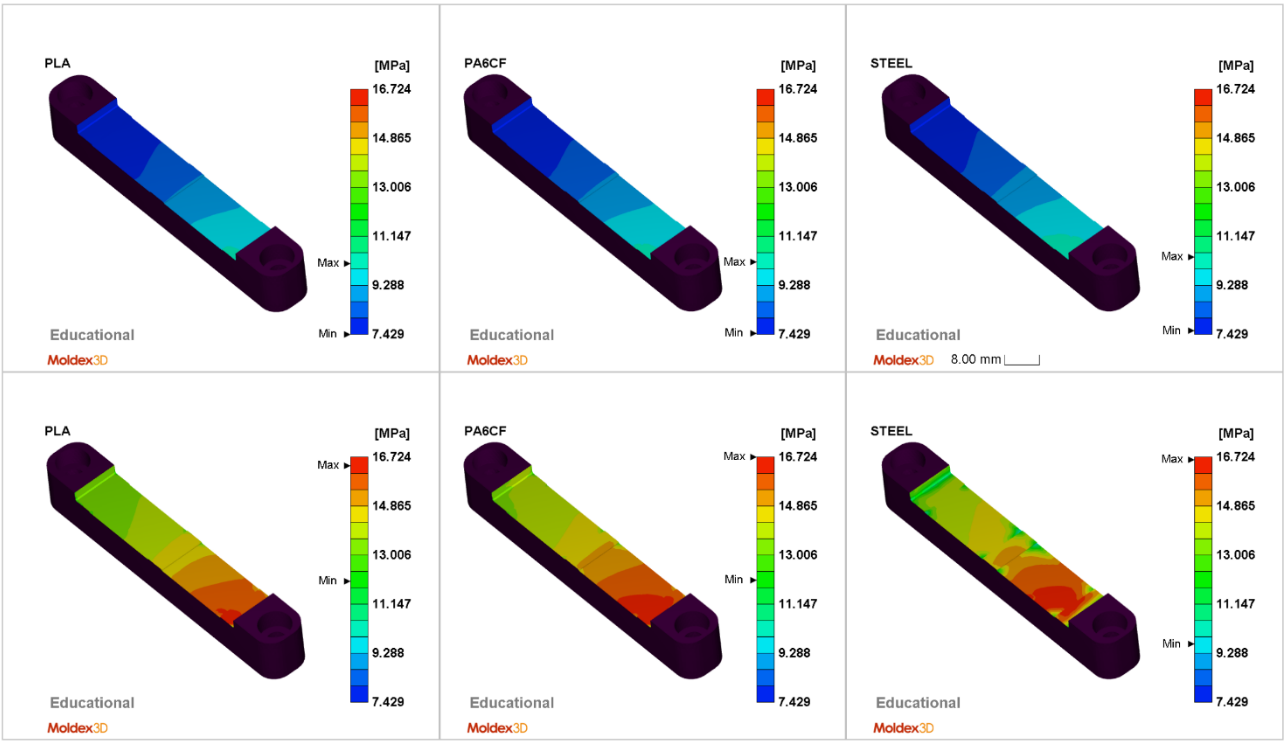

Figure S7. Insert pressure prediction at the end of filling step (EOF) from Moldex3D simulation software with insert made of PLA, PA6CF, and steel and injection molding PP (upper plots) and LDPE (lower plots).

Table S2. Moldex 3D simulation data at the end of filling (EOF), end of cooling (EOC), and after opening and eject (Open).

Injected material	Insert material	Max T EOF (°C)	Max T EOC (°C)	Max T Open (°C)	Max P EOF+EOP (MPa)
LDPE	PLA_TT	99	70	63	16
LDPE	PA6CF_TT 2	97	65	57	17
LDPE	Steel	45	34	33	17
PP	PLA_TT	144	101	88	10
PP	PA6CF_TT 2	140	94	79	10
PP	steel	58	41	39	10

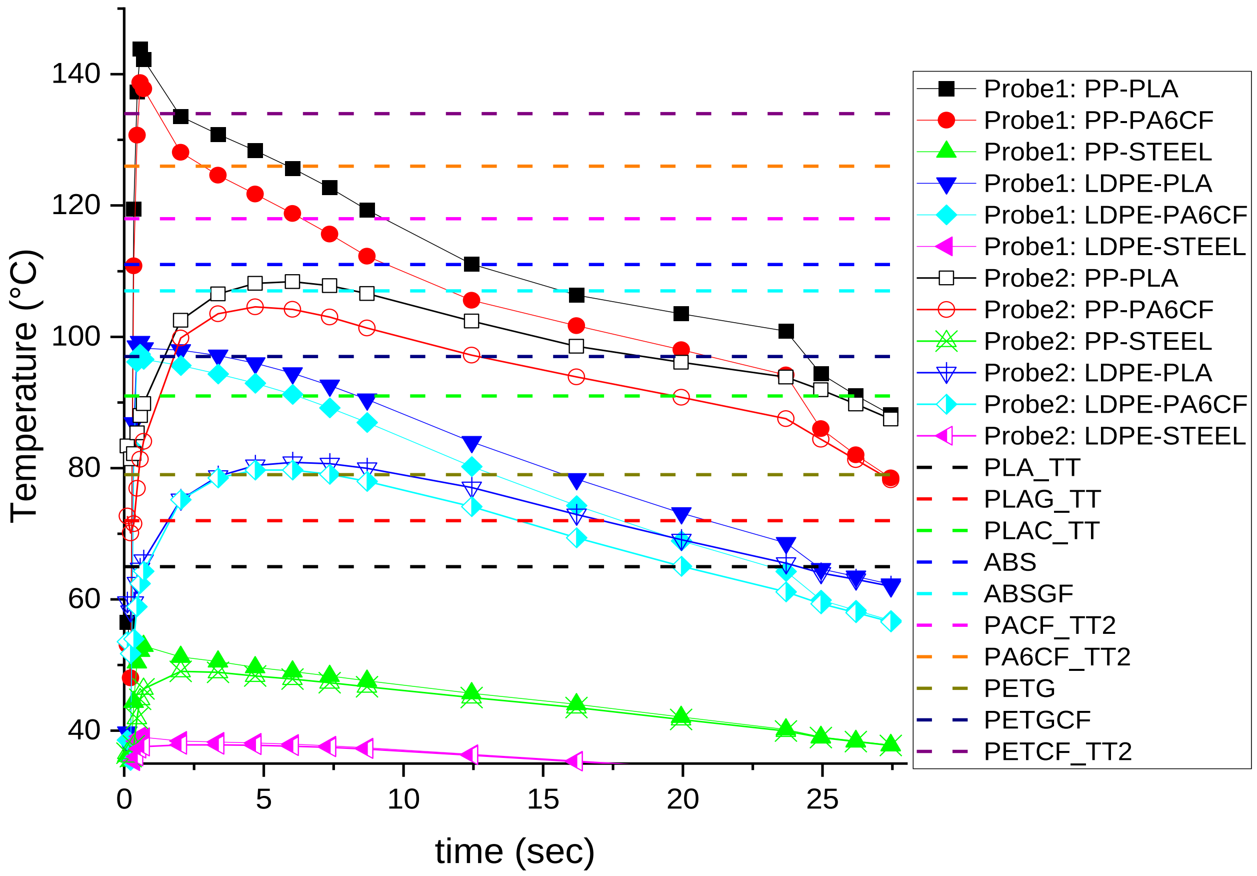

Figure S8. Insert temperature prediction during the entire process from Moldex3D simulation software with insert made of PLA, PA6CF, and steel and injection molding PP and LDPE. Probe 1 is on the surface corresponding to the maximum temperature point and Probe 2 is under the Probe 1 at a depth of 0.5mm. The dotted lines correspond to the HDT-A temperatures obtained with the various materials.

Table S3. Insert temperature prediction from Moldex3D simulation software compared to HDT-A.

Material	HDT-A	LDPE avg T	Diff. avg T	LDPE probe2	Diff. probe2	PP avg T	Diff. avg T	PP probe2	Diff. probe2
PLA_TT	65	94	29	81	16	128	63	108	43
PLAG_TT	72	94	22	81	9	128	56	108	36
<u>PLAC_TT</u>	91	92	1	80	-11	124	33	104	13
ABS	111	94	-17	81	-30	128	17	108	-3
ABSGF	107	94	-13	81	-26	128	21	108	1
<u>PACF_TT2</u>	118	92	-26	80	-38	124	6	104	-14
<u>PA6CF_TT2</u>	126	92	-34	80	-46	124	-2	104	-22
PETG	79	94	15	81	2	128	49	108	29
<u>PETGCF</u>	97	92	-5	80	-17	124	27	104	7
<u>PETCF_TT</u>	134	92	-42	80	-54	124	-10	104	-30

Materials were classified as unreasonable if HDT-A was more than 10 °C below the simulated temperature range, risky if within ±10 °C, and reasonable if at least 10 °C above the simulated temperature. Both the average and maximum simulated temperatures were considered to account for thermal gradients within the insert.

The <u>underlined materials</u> are those with the highest thermal conductivity characteristics.

Videos acquired with thermo camera

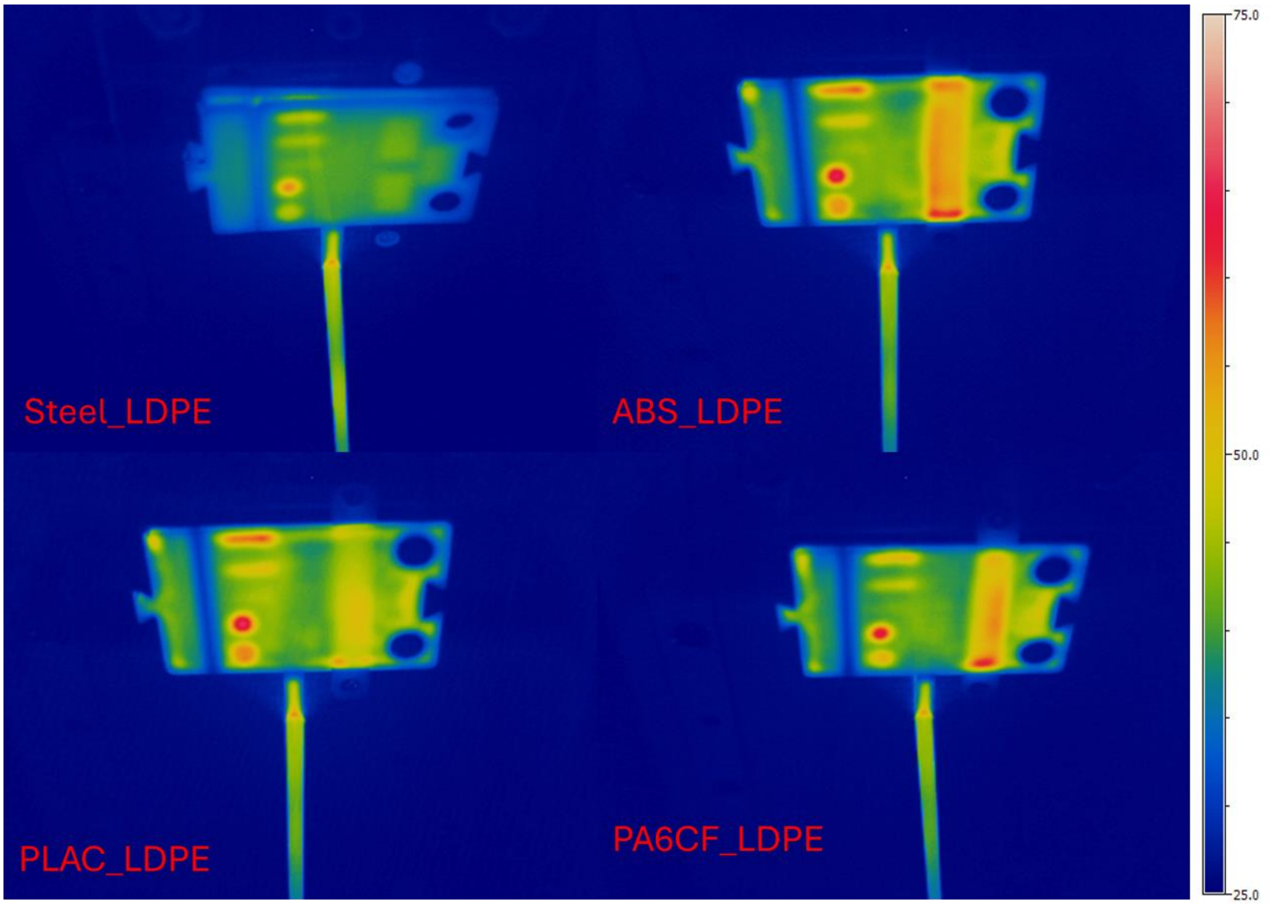

Figure S9. Thermo-camera pictures of objects before ejection from the mold.

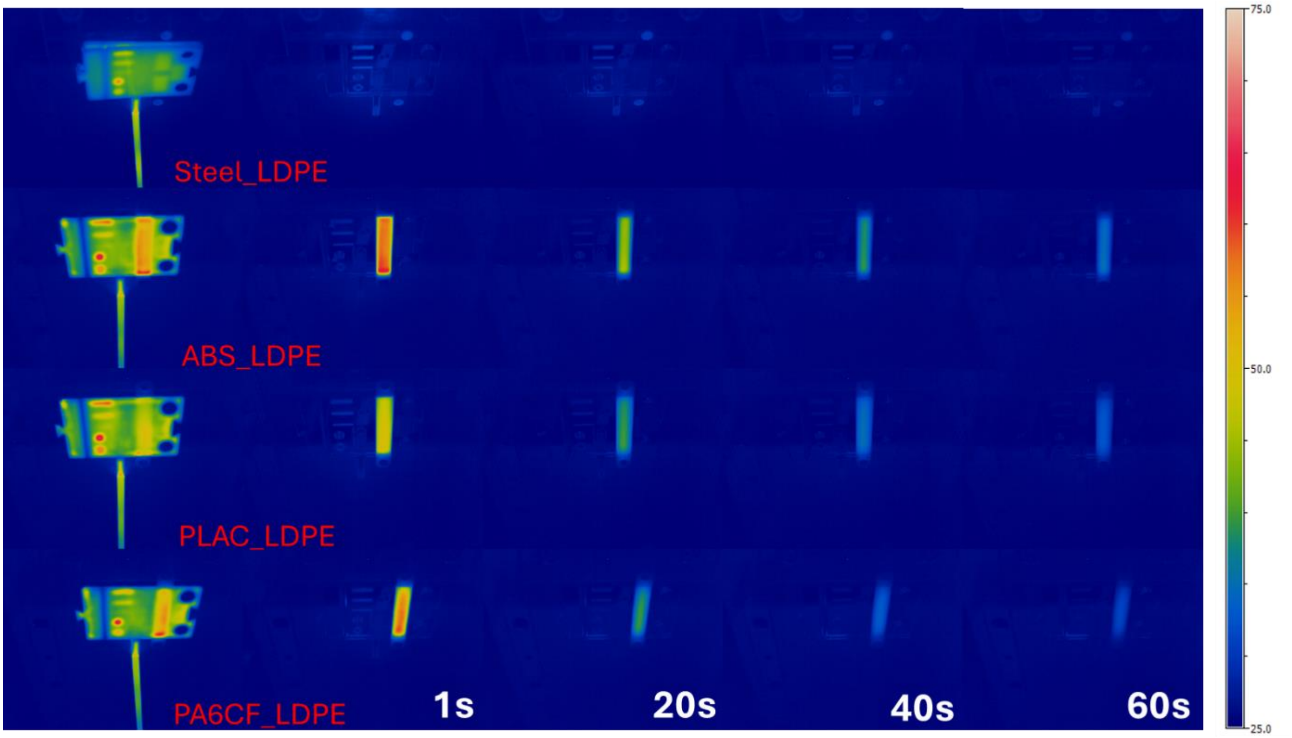

Figure S10. Thermo-camera pictures of inserts after ejection.

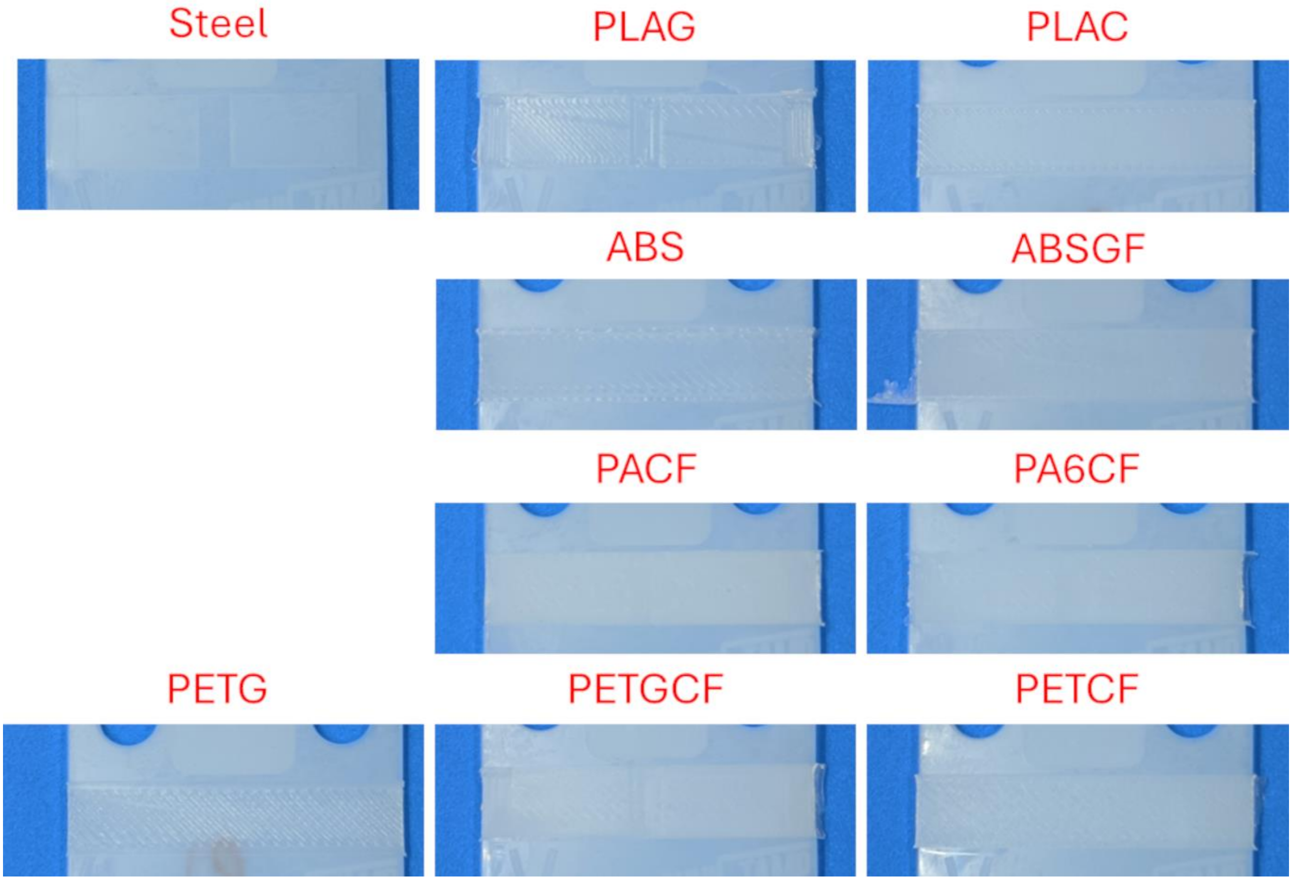

Figure S11. Visual comparisons of the parts of the LDPE injection-molded objects that correspond to the inserts.
